# The impact of exclusion processes on angiogenesis models

**DOI:** 10.1007/s00285-018-1214-1

**Published:** 2018-03-06

**Authors:** Samara Pillay, Helen M. Byrne, Philip K. Maini

**Affiliations:** Mathematical Institute, University of Oxford, Woodstock Road, Oxford, OX2 6GG UK

**Keywords:** Angiogenesis, Cellular automaton, Multiscale modeling, Partial differential equations, Volume exclusion, Chemotaxis, 35K57, 35Q92, 60J70, 92C17

## Abstract

Angiogenesis is the process by which new blood vessels form from existing vessels. During angiogenesis, tip cells migrate via diffusion and chemotaxis, new tip cells are introduced through branching, loops form via tip-to-tip and tip-to-sprout anastomosis, and a vessel network forms as endothelial cells, known as stalk cells, follow the paths of tip cells (a process known as the snail-trail). Using a mean-field approximation, we systematically derive one-dimensional non-linear continuum models from a lattice-based cellular automaton model of angiogenesis in the corneal assay, explicitly accounting for cell volume. We compare our continuum models and a well-known phenomenological snail-trail model that is linear in the diffusive, chemotactic and branching terms, with averaged cellular automaton simulation results to distinguish macroscale volume exclusion effects and determine whether linear models can capture them. We conclude that, in general, both linear and non-linear models can be used at low cell densities when single or multi-species exclusion effects are negligible at the macroscale. When cell densities increase, our non-linear model should be used to capture non-linear tip cell behavior that occurs when single-species exclusion effects are pronounced, and alternative models should be derived for non-negligible multi-species exclusion effects.

## Introduction

**Fig. 1:**
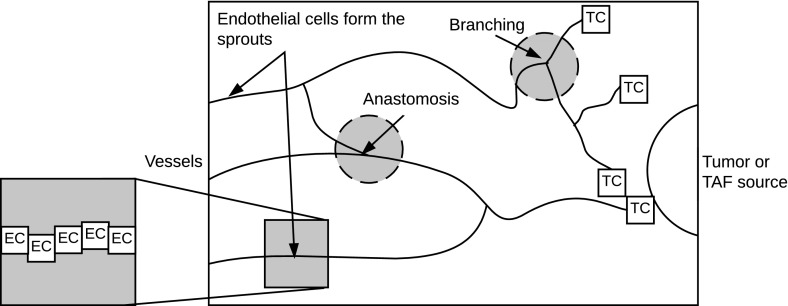
A schematic of angiogenesis. Tip cells (TCs) move via chemotaxis in response to a tumor angiogenic factor (TAF) to direct sprout movement. Endothelial cells (ECs), known as stalk cells, follow the paths of TCs and proliferate and elongate behind the leading TCs. Sprouts connect through anastomosis and new TCs are introduced through branching

Angiogenesis is the process by which new blood vessels develop from existing blood vessels. Angiogenesis is important in developmental processes, such as embryogenesis, in wound healing and in pathological conditions, including cancer. Solid tumors initiate angiogenesis by secreting tumor angiogenic factors (TAFs) such as vascular endothelial growth factor (VEGF) (Klagsbrun and Moses 1999; Folkman and Klagsbrun 1987; Carmeliet and Jain 2011). These TAFs diffuse from the tumor towards the vasculature, creating a spatial concentration gradient between the two. On reaching the nearby blood vessels, the TAFs stimulate the endothelial cells lining the vessels to degrade the basement membrane (Klagsbrun and Moses 1999; Carmeliet and Jain 2011; Potente et al. 2011), and migrate via chemotaxis up spatial gradients towards the TAF source (Ausprunk and Folkman 1977). Tip cells (TCs) at the head of a sprout use filopodia to sense environmental cues and direct the movement of the sprout (Potente et al. 2011; Carmeliet and Jain 2011; Benitez and Heilshorn 2013) (see Fig. 1). Endothelial cells (ECs), also known as stalk cells (Carmeliet and Jain 2011; Potente et al. 2011), behind the TCs, follow the TCs and proliferate and elongate to form longer sprouts (Klagsbrun and Moses 1999; Potente et al. 2011; Carmeliet and Jain 2011), a process that has been termed a “snail-trail”. Sprouts connect to form loops through anastomosis (Carmeliet and Jain 2011; Potente et al. 2011), which is essential for establishing blood flow. New TCs are created via branching, a process stimulated by TAFs (Carmeliet and Jain 2011) and mediated by Delta-Notch signaling (Carmeliet and Jain 2011; Potente et al. 2011).

The corneal assay, in which a TAF source is implanted into the cornea of a mouse or small animal, is widely used to study angiogenesis in vivo as the cornea is avascular (Auerbach et al. 2003). In the cornea, sprouting occurs from vessels located at the limbus, situated approximately 1–2 mm from the TAF source (Muthukkaruppan et al. 1982; Gimbrone et al. 1974), marking the separation between the cornea and white of the eye. Since the cornea is thin, approximately 100 $\upmu$m thick in mice (Schulz et al. 2003; Zhang et al. 2013), angiogenesis in the corneal assay is typically modeled as a two-dimensional process. As the vascular network approaches the tumor, experimental results in the corneal assay indicate that the vessel density, in particular, the TC density, increases, a phenomenon known as the brush-border effect (Muthukkaruppan et al. 1982).

The importance of angiogenesis in biology is mirrored by the numerous mathematical models that have been developed to increase understanding of the processes by which it is regulated [for details, see the reviews of Mantzaris et al. (2004) and Scianna et al. (2013), and references therein]. In this work, we focus on systematically deriving continuum snail-trail models from discrete, agent-based models, and, therefore, review only literature directly relevant to such approaches. Continuum models, known as snail-trail models (Balding and McElwain 1985; Byrne and Chaplain 1995), describe the spatio-temporal evolution of the TC and EC densities in the corneal assay using partial differential equations (PDEs). They are typically one-dimensional and account for two-dimensional effects by considering vessel densities averaged in the direction perpendicular to that in which the vascular front propagates (Balding and McElwain 1985; Byrne and Chaplain 1995; Connor et al. 2015). Other continuum models focus on the TC population only (Anderson and Chaplain 1998; Chaplain 1995, 1996, 2000; Chaplain and Stuart 1993). Agent-based models, which account for the behavior of individual cells (Anderson and Chaplain 1998; Chaplain 2000; Stéphanou et al. 2005), cell-cell interactions and sub-cellular processes such as signaling (Bentley et al. 2008, 2009; Jakobsson et al. 2010), have been developed to reveal emergent behavior at the macroscale. Hybrid models (Tong and Yuan 2001; Harrington et al. 2007), which combine aspects of both continuum and discrete models, have been developed to integrate dynamics that operate across multiple scales.

Recently, several authors have derived continuum models from stochastic microscale (Pillay et al. 2017) or mesoscale models (Spill et al. 2015; Bonilla et al. 2014; Terragni et al. 2016) using a mean-field approach, in order to facilitate a more complete understanding of angiogenesis, especially with regards to how microscale processes (e.g. cell-level behavior that influences vessel structure) affect population-behavior (nutrient and drug transport). For example, Bonilla et al. (2014) used a stochastic, mesoscale framework to derive an integro-differential PDE for the TC population. Spill et al. (2015) used a mesoscale, mean-field approach to reproduce the snail-trail model of Byrne and Chaplain (1995) (hereafter refered to as BC). In contrast, Pillay et al. (2017) used a microscale mean-field approach to show that the BC model qualitatively captures angiogenesis in two dimensions but must be modified by a scaling factor to accurately account for cell densities.

Continuum models that neglect volume exclusion, the reduced space available to cells as a result of their finite size, are generally linear and, therefore, more amenable to analysis. Volume exclusion has been neglected in many continuum models of angiogenesis (Balding and McElwain 1985; Byrne and Chaplain 1995; Connor et al. 2015; Spill et al. 2015; Pillay et al. 2017; Chaplain and Stuart 1993; Chaplain 1995, 1996), while discrete models account for the volume of cells (Anderson and Chaplain 1998; Chaplain 2000; Bonilla et al. 2014; Tong and Yuan 2001; Bentley et al. 2008, 2009; Jakobsson et al. 2010) naturally. In Balding and McElwain (1985), Byrne and Chaplain (1995) and Connor et al. (2015), volume exclusion from both the TC and EC populations is neglected. Only the TC population is modeled in Anderson and Chaplain (1998), Chaplain (1995, 1996, 2000) and Chaplain and Stuart (1993) and, as a result, the impact of volume exclusion on interactions between TCs and ECs, and on TC motility is neglected. Spill et al. (2015) incorporate TC crowding effects in the diffusive motion, but neglect crowding effects due to ECs. In Pillay et al. (2017), TC and EC volume exclusion is neglected in order to derive, from a lattice-based microscale model, a model that is comparable to the BC model.

Simple exclusion processes that account for volume exclusion are frequently used to model cell movement in discrete models (see Penington et al. 2011; Simpson et al. 2007, 2009a, b, 2010; Callaghan et al. 2006 and references therein): they allow at most one motile agent to occupy a lattice site and, thereby, allow for biologically realistic interactions. Symmetric exclusion processes, such as lattice-based, unbiased random walks, can be described at the macroscale by a linear diffusion equation (Liggett 1999; Simpson et al. 2009a, b, 2010). While agents interact with each other, the interactions cancel due to the symmetry of the system and, therefore, do not appear in the macroscopic description (Penington et al. 2011; Simpson et al. 2009a). In contrast, asymmetric exclusion processes (see Penington et al. 2011 and references therein), such as biased random walks, or multi-species exclusion processes (Simpson et al. 2009a), give rise to non-linear advection-diffusion equations. For example, Simpson et al. (2009a) derived a system of non-linear advection-diffusion equations describing asymmetric exclusion processes (biased random walks), which can be interpreted in terms of fluxes for interacting subpopulations. Dyson and Baker (2015) considered two interacting subpopulations in an off-lattice framework and then used a mean-field approximation to derive non-linear advective-diffusive equations for each population. They also explored the importance of volume exclusion in migratory cell models, and found that volume exclusion effects are most important in biased movement, such as chemotactic migration. In these cases, continuum models that account for exclusion effects more accurately capture individual-based simulations than those which do not (Dyson and Baker 2015). Corrections to mean-field descriptions (based on a moment dynamics approach) for a variety of exclusion-process-based models have been investigated by Simpson and Baker (2011).

In this work, we aim to determine how exclusion processes, specifically TC and EC volume exclusion, affect angiogenesis. To this end, we extend the framework in Pillay et al. (2017) by explicitly incorporating cell volume. We systematically derive a one-dimensional PDE model (using a mean-field approximation) from a two-dimensional cellular automaton (CA) model of corneal angiogenesis incorporating TC migration, branching and anastomosis (loop formation) as well as volume exclusion in the motility and branching mechanisms. We derive two models, one in which TCs do not interact with ECs (Model 1), so that only TC volume exclusion is active, and a second model (Model 2) which incorporates both TC and EC volume exclusion. The resulting PDEs are non-linear in the diffusive, chemotactic, branching and anastomosis terms. By comparing Models 1 and 2, we can distinguish the macroscale effects of TC volume exclusion and EC volume exclusion. We also compare the modified BC model (see Pillay et al. 2017) to averaged simulation results from the CA model to determine whether a snail-trail model that is linear in the diffusive, chemotactic and branching terms can account for macroscale volume exclusion effects.

The remainder of this work is structured as follows. In Sect. 2, we introduce our CA model. In Sect. 3, we derive one-dimensional PDE models using transition probabilities associated with the CA model, a mean-field approximation and column-averaged difference equations for cell occupancy. In Sect. 4, we discuss the structure of these non-linear continuum models. In Sect. 5, we evaluate the validity of the mean-field approximation used to derive the continuum models. Based on these comparisons, we conclude that we should estimate parameters in the continuum models that arise from processes that lead to correlation effects by fitting the models to synthetic data from averaged CA simulations. In Sect. 6, we compare solutions of our PDE models and the BC model with averaged CA simulation results to distinguish macroscale volume exclusion effects and determine whether linear continuum models can account for these effects. We close with a discussion of our key findings in Sect. 7.

## Cellular automaton

We use a regular two-dimensional, square lattice to represent the cornea. The lattice sites are indexed by (*i*, *j*) with $i,j, \in \mathbb {Z}^+$ ($0 \le i,j \le R$). The lattice spacing represents the diameter of a cell. The limbus (border between the cornea and white of the eye where blood vessels are situated) and TAF source are situated at $i=0$ and $i=R$, respectively. The variable $\bar{K}$ represents discrete time in our CA model. The microscopic and macroscopic variables are related via $x_i =i h,y_j = j h, Rh =1, t = \bar{K} \tau$, where *h* represents the lattice spacing and $\tau$ represents the time step.

We present our CA model in non-dimensionalized units, where we have rescaled space with distance from the limbus to TAF source *L*, and time with the TAF diffusion timescale, $L^2/D_{TAF}$, where $D_{TAF}$ is the TAF diffusion coefficient. We apply a linear TAF field (non-dimensional) $c(x,y,t) = x \in [0,1]$ (discretized as $c_{i,j}=ih$), derived assuming a diffusion-dominated quasi-steady-state approximation (see Pillay et al. 2017 for details). We use this simplified TAF field as it allows us to focus on cell dynamics. As there are two cell phenotypes involved in angiogenesis, migratory TCs and ECs (stalk cells) which follow TCs, we define two agent types on our lattice: active TCs and passive ECs.

We develop two CA models: for Model 1 TCs only interact with other TCs via tip-to-tip anastomosis and TC volume exclusion; for Model 2, TCs also interact with ECs via tip-to-sprout anastomosis and EC volume exclusion. Comparing these models will enable us to distinguish the effects of TC volume exclusion and EC volume exclusion. We define the state space (permitted occupancy of a lattice site), *A*, in each model as follows. In Model 1, $A=TC \times EC = \{0, 1\} \times \{0, Z\}$ where 0 indicates that the site is unoccupied by the specified species, 1 indicates a site is occupied by a TC and *Z* indicates the site is occupied by integer values of ECs. In Model 2, $A=\{0, 1, 2\}$, where 0 indicates a vacant site, 1 indicates a TC is present and 2, that an EC is present.

The positions of TCs are updated using a random sequential update. At the beginning of the $\bar{K}$th discrete time step, a TC is selected independently at random from the $\bar{N}$ TCs on the lattice. The chosen TC at site (*i*, *j*) is then given the opportunity to move with probability $P_m \in [0,1]$. The TC may move to sites within its von Neumann neighborhood $(i\pm 1,j\pm 1)$, and may undergo exclusion or anastomosis (see Sect. 2.1 for CA rules), based on the probabilities $P^{x\pm }_{i,j}$ and $P^{y\pm }_{i,j}$ (see Eqs. (1) and (2) below). Specifically, $P_m$ determines the probability with which a chosen TC may move in a given time step, $P^{x\pm }_{i,j}$ and $P^{y\pm }_{i,j}$ determine the directional probability of the move, and if the target site is occupied, then the move is aborted (exclusion). If a TC move is allowed (due to vacancy of the target site or an anastomosis event), then an EC is left behind at the original TC site, creating a snail-trail of vessels. Following $\bar{N}$ sequential motility attempts ($\bar{N}$ TCs are selected independently at random from the TCs on the lattice and each is given the opportunity to move), during the same discrete time step $\bar{K}$, $\bar{N}$ branching attempts are made by selecting $\bar{N}$ TCs one at a time at random. These agents are given the opportunity to branch, with daughter TCs placed at sites $(i,j\pm 1)$ with probability $P_b \in [0,1]$ provided the target sites are unoccupied. ECs are not actively updated in the CA model; rather, their evolution is determined by TC movement.

### Movement and anastomosis

The movement probabilities $P^{x\pm }$ and $P^{y\pm }$ describe a biased random walk and are defined as follows

1 $$\begin{aligned} P^{x\pm }_{i,j} = \frac{1\pm k\left( c_{i+1,j}-c_{i-1,j}\right) }{4}, \end{aligned}$$

2 $$\begin{aligned} P^{y\pm }_{i,j} = \frac{1\pm k\left( c_{i,j+1}-c_{i,j-1}\right) }{4}. \end{aligned}$$

In particular, $P^{x\pm }$ and $P^{y\pm }$ consist of a random motion term (1 / 4), and a term that biases TC motion in the direction of increasing TAF concentration through a central difference approximation to the TAF gradient at the location of the TC. The constant parameter $k>0$ scales the chemotactic response of TCs (and is also chosen such that the probabilities lie between 0 and 1). For the linear TAF profile considered here, $c_{i,j} = c_i$, and therefore $P^{y\pm } =\frac{1}{4}$.

**Fig. 2:**
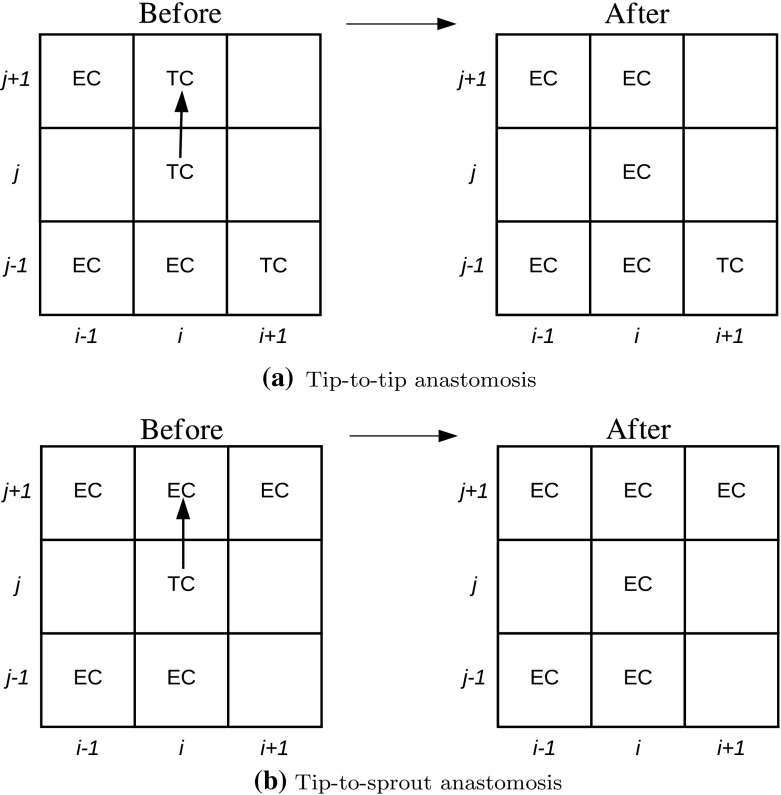
**a** Tip-to-tip anastomosis (Model 1 and 2): If a tip cell (TC) at site (*i*, *j*) moves to site $(i,j+1)$, occupied by another TC, then both TCs are removed from the simulation and ECs are placed at sites (*i*, *j*) and $(i,j+1)$ (due to TC movement). **b** Tip-to-sprout anastomosis (Model 2): If a TC at site (*i*, *j*) moves to site $(i,j+1)$, occupied by an EC, then the TC is removed from the simulation, the EC at site $(i,j+1)$ remains and an additional EC is placed at site (*i*, *j*) (due to TC movement)

Anastomosis and exclusion (aborted TC moves due to occupancy) are processes that are linked in our model. Anastomosis is the formation of loops that are created when TCs connect with other TCs or ECs. We assume that anastomosis annihilates TCs (as ECs are responsible for lumen formation once connections form (Potente et al. 2011; Carmeliet and Jain 2011; Blanco and Gerhardt 2013)). Tip-to-tip anastomosis occurs if a TC at site (*i*, *j*) moves to a neighboring site occupied by another TC. Both TCs are removed from the simulation and replaced with ECs (see Fig. 2a). Tip-to-sprout anastomosis (defined in Model 2 only) occurs if a TC at (*i*, *j*) moves to a neighboring site occupied by an EC. The TC is replaced by an EC and the original EC remains (see Fig. 2b). Implicit in the aforementioned description is the assumption that when an anastomosis event occurs, TC moves are not aborted due to occupancy of the target site. We implement anastomosis using two variables, $a_n$ and $a_e$, defined as the probability of a tip-to-tip and tip-to-sprout anastomosis event occurring, respectively. Thus, $1-a_n$ and $1-a_e$ are the probabilities that TC volume exclusion and EC volume exclusion occur, respectively. To be specific, if a TC attempts to move to a site that is occupied by a TC or EC, an anastomosis event occurs with probability $a_n$ or $a_e$, respectively. Otherwise, the move is aborted. This differs from the CA framework in Pillay et al. (2017) where anastomosis events occurred every time a TC encountered another cell.

In all cases, once a TC moves from a site, an EC is left behind at that site creating the trail of vessels associated with the snail-trail. Further, we prohibit self-loops (a TC anastomosing with an EC in its own sprout, i.e. an EC it has left behind) during tip-to-sprout anastomosis (see Pillay et al. 2017 for details). In such cases, the TC move is aborted (EC volume exclusion). This outcome differs from Pillay et al. (2017) where such moves were permitted without causing anastomosis.

### Branching

**Fig. 3:**
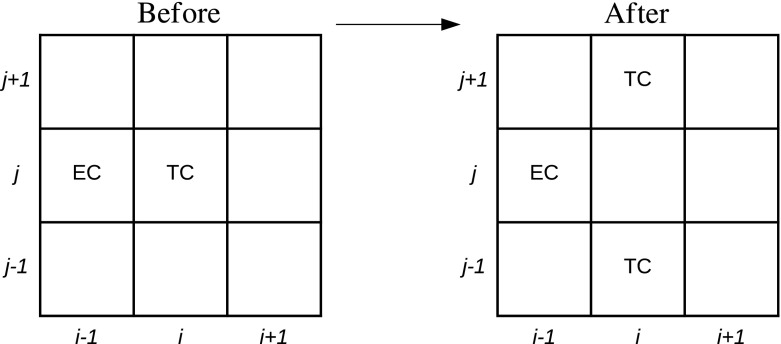
A tip cell (TC) at site (*i*, *j*) may branch during a time step by placing daughter TCs at $(i,j\pm 1)$, provided those sites are unoccupied by TCs (Models 1 and 2), or by ECs (Model 2)

We assume that branching occurs in the direction perpendicular (*y*-direction) to that of propagation of the TCs (towards the TAF source, in the *x*-direction) as in Pillay et al. (2017). Since branching is stimulated by TAFs (Carmeliet and Jain 2011), we assume that the probability with which a TC at site (*i*, *j*) branches, $P_b$, increases linearly with TAF concentration as follows: $P_b= P_p c \in [0,1]$ ($P_p$ is a model parameter, assumed constant, that scales the TAF concentration and ensures that $P_b \le 1$). This functional form of the branching probability models the brush-border effect (Muthukkaruppan et al. 1982), i.e. an increase in TC density as the TC front approaches the TAF source. A TC at site (*i*, *j*) branches by placing daughter TCs at sites $(i,j\pm 1)$ provided those sites are unoccupied (by TCs in Model 1; by TCs or ECs in Model 2) and the TC at site (*i*, *j*) is removed (see Fig. 3), creating the patterning associated with Delta-Notch signaling (Potente et al. 2011; Carmeliet and Jain 2011). We assume that ECs are not created through branching. Our branching configuration is consistent with that used in Pillay et al. (2017), and we note that other configurations are possible.

### Initial conditions, boundary conditions and cell occupancy

Our initial conditions represent sprouting from the limbus following the initiation of angiogenesis (i.e. once TCs become motile). We place TCs along $i=0$ at alternating lattice sites i.e. 

3 $$\begin{aligned} \mathrm {TCs \; placed \; at \;} (i=0, j=1, 3, \ldots , R-1). \end{aligned}$$

ECs are created once TCs begin to move, and thus, initially, there are no ECs on the lattice. In practice, TAF (VEGF) and Delta-Notch signaling (Carmeliet and Jain 2011; Potente et al. 2011; Blanco and Gerhardt 2013) mediate TC selection at the limbus. We prescribe the initial TC spacing in our model, as in Pillay et al. (2017).

TCs are not permitted to cross lattice boundaries (no flux boundary conditions). This is justified by the fact that TCs cannot cross the boundaries of the cornea, and at the TAF source ($i=R, 0\le j\le R$), our model does not account for interactions between TCs and the TAF source/tumor fragment.

We summarize Models 1 and 2, and the corresponding variables and rules, in Table 1. The CA algorithm is outlined in Appendix B.

**Table 1:** Summary of variables and rules used in CA Models 1 and 2

Model	Volume exclusion	Anastomosis	Max occupancy	$a_n$	$a_e$
1	TC	Tip-to-tip	1 TC, ECs $\ge 1$	[0,1]	N/A
2	TC, EC	Tip-to-tip, tip-to-sprout	1 TC or EC	[0,1]	[0,1]

### Ensemble averages

In order to relate the discrete models to a continuum, macroscopic description, we average the TC and EC occupancy of site (*i*, *j*) over *M* realizations of the discrete model at discrete times $1, 2,\ldots , \bar{K}$. We introduce averaged quantities $n_{i,j}(\bar{K})$ and $e_{i,j}(\bar{K})$, respectively, where

4 $$\begin{aligned} n_{i,j}(\bar{K}) = \frac{1}{M} \sum _{m=1}^M n^m_{i,j} (\bar{K}), \quad e_{i,j}(\bar{K})=\frac{1}{M} \sum _{m=1}^M e^m_{i,j}(\bar{K}), \quad 0\le i,j \le R. \end{aligned}$$

Here $n^m_{i,j}(\bar{K})$ and $e^m_{i,j}(\bar{K})$ are the TC and EC occupancies at site (*i*, *j*) after $\bar{K}$ discrete time steps of the *m*th realization of the discrete model. The TC occupancy is defined as $n^m_{i,j}(\bar{K}) \in \{0,1\}$ (either 1 (occupied) or 0 (unoccupied)). The EC occupancy in Model 1 is defined as $e^m_{i,j}(\bar{K}) \in \{0,Z\}$, where *Z* indicates positive integer occupancy, while in Model 2 it is defined as $e^m_{i,j}(\bar{K}) \in \{0,1\}$. We also define column averaged TC and EC occupancies, $N_i(\bar{K})$ and $E_i(\bar{K})$, as follows:

5 $$\begin{aligned} N_i(\bar{K})= & {} \frac{1}{M (R+1)} \sum _{m=1}^M \sum _{j=0}^{R} n^m_{i,j}(\bar{K}), \end{aligned}$$

6 $$\begin{aligned} E_i(\bar{K})= & {} \frac{1}{M (R+1)} \sum _{m=1}^M \sum _{j=0}^{R} e^m_{i,j}(\bar{K}), \end{aligned}$$

where *R* is the number of columns on the lattice.

## The continuum model

We now derive mean-field equations for the expected TC and EC densities in Models 1 and 2. For Model 2, we derive a PDE model for which $a_e=1$ (tip-to-sprout anastomosis occurs with probability 1, unless a self-loop is prohibited). In Sect. 5.2, we will estimate $a_e$ by fitting the Model 2 PDEs to averaged CA simulation results to account for prohibited self-loops. Thus, we retain $a_e$ in the derivation below. We distinguish between Models 1 and 2 (see Table 1 for model definitions) in the derivation by introducing a variable, *B*, such that

7 $$\begin{aligned} B={\left\{ \begin{array}{ll} 0, &{} \text {Model 1},\\ 1, &{} \text {Model 2}. \end{array}\right. } \end{aligned}$$

The variable *B* allows us to control the volume exclusion incorporated in the branching term (EC volume exclusion in Model 2, only TC volume exclusion in Model 1), as well as either include or exclude tip-to-sprout anastomosis in Models 2 and 1, respectively. We derive continuum models by formulating difference equations that relate $n_{i,j}(\bar{K}+1)$ and $e_{i,j}(\bar{K}+1)$, the average TC and EC occupancies, respectively, at site (*i*, *j*) at time $\bar{K}+1$, defined in Eq. (4), to the average occupancies at site (*i*, *j*) at time $\bar{K}$. Defining $\delta n_{i,j}$ and $\delta e_{i,j}$ as

8 $$\begin{aligned} \delta n_{i,j}= & {} n_{i,j}(\bar{K}+1)-n_{i,j}(\bar{K}), \end{aligned}$$

9 $$\begin{aligned} \delta e_{i,j}= & {} e_{i,j}(\bar{K}+1)-e_{i,j}(\bar{K}), \end{aligned}$$

we formulate difference equations for the TC and EC occupancies as follows:10 and11We have formulated Eqs. (10) and (11), which describe how TC and EC occupancies, respectively, change during a time step, using a mean-field approximation i.e assuming that the occupancy of lattice sites is independent.

The first line on the right-hand side of Eq. (10) represents TC movement from neighboring sites $(i\pm 1,j)$ and $(i,j\pm 1)$ into site (*i*, *j*). When interpreting this term, it is convenient to decompose $(1-n_{i,j} - Ba_e e_{i,j})$ as $([1-(1-a_n) n_{i,j}] - a_n n_{i,j} - B a_e e_{i,j})$. The term $(1-(1-a_n) n_{i,j})$ describes movement from neighboring sites into (*i*, *j*) where TC volume exclusion occurs with probability $(1-a_n)$ if site (*i*, *j*) is occupied by a TC. The penalty terms $-a_n n_{i,j}$ and $- B a_e e_{i,j}$ represent tip-to-tip and tip-to-sprout anastomosis (as in Pillay et al. 2017), respectively, which occur with probability $a_n$ and $a_e$, respectively. In particular, if a TC moves from neighboring sites into (*i*, *j*), which is occupied by a TC or EC, then a tip-to-tip or tip-to-sprout anastomosis event occurs, respectively, and the TC is removed (see anastomosis rules in Sect. 2.1). Thus, the term $(1-n_{i,j} - Ba_e e_{i,j})$ implies that a TC can only move into site (*i*, *j*) and remain a TC if that site is vacant.

Anastomosis and volume exclusion affect TC movement out of a site differently. TCs move out of a site into neighboring sites where TC volume exclusion occurs with probability $1-a_n$ [see lines 2 and 3 of Eq. (10)] if the target site is occupied by a TC. If $a_n=1$, then TCs are always allowed to move out of a site, and the outcome depends on the occupancy of the target site.

Branching incorporates volume exclusion; it can only occur if neighboring sites are unoccupied by TCs (Model 1 and 2) or ECs (Model 2) [see lines 4–6 of Eq. (10)]. The branching terms in Eq. (10) represent branching into site (*i*, *j*) from a TC located at $(i,j\pm 1)$ (source terms), and the loss of a TC from site (*i*, *j*) that branches into neighboring sites (sink term).

ECs are created by TC movement out of a site and via tip-to-tip anastomosis, which is represented in Eq. (11). Thus, the source terms in Eq. (11) are the positive counterparts of sink terms in Eq. (10) (representing TC movement out of a site and tip-to-tip anastomosis). Tip-to-sprout anastomosis does not affect the EC population.

We formulate the difference equations in terms of column averages, so that the resulting PDEs will be one-dimensional. Therefore, we multiply both sides of Eqs. (10) and (11) by $1/(R+1)$ and sum over the column index *j*. We then apply a mean-field approximation for column averages (see Simpson et al. 2009a; Pillay et al. 2017), by approximating products of occupancy terms by products of column averages. Under these approximations and given that $P^{y\pm }_{i,j}=P^{y\pm }_i$ (TAF concentration is linear in *x*), the column-averaged difference equations for the TCs and ECs are, respectively,12and13When considering column averages for motility terms (excluding anastomosis), movements in *y* do not affect the TC density and, thus, only TC motility terms between columns remain [see Eq. (12)]. By contrast, for anastomosis, movement within a column may affect the TC density through TC annihilation and, therefore, movements within and between columns are accounted for. Given that branching occurs in the *y*-direction, the branching terms describe processes that are localized within a single column; they are derived by summing the branching terms in Eq. (10) over *j* and applying a mean-field approximation for column averages.

ECs are created either when a TC leaves a site or through tip-to-tip anastomosis. Thus, in Eq. (13), TC movement in the *y*-direction (i.e. within a column) is retained, and the tip-to-tip anastomosis term is the positive counterpart of the corresponding term in Eq. (12).

We note that as the initial TC occupancies in the CA depend on *j* [see Eq. (3)], our column-averaging procedure is not exact, and assumes further mean-field approximations as discussed. However, we will show in Sects. 5 and 6, when comparing our PDE solutions to averaged simulation results, that when the mean-field approximations hold, our approximations are reasonable.

We now expand the dependent variables, *N*, *E* and *c*, in Eqs.  (12) and (13) in a Taylor series about the site *i* in order to relate the column-averaged difference equations to a continuum model. By setting $x_i \rightarrow x, N_{i}(\bar{K}) \rightarrow N(x,t), E_{i}(\bar{K})\rightarrow E(x,t)$, dividing the resulting expressions by $\tau$, which relates the discrete and continuum time scales ($t = \tau \bar{K}$), taking the limit as $\tau \rightarrow 0$, and neglecting terms of $O(h^4)$ and higher, we can relate our difference equations to the following PDEs for *N*(*x*, *t*) and *E*(*x*, *t*):

14 $$\begin{aligned} \frac{\partial N}{\partial t}= & {} D ( 1-a_n N-B a_eE) \frac{\partial ^2 N}{\partial x^2} \nonumber \\&-\chi \left( \frac{\partial }{\partial x} \left( N(1-N) \frac{\partial c}{\partial x}\right) +a_n N \frac{\partial N}{\partial x} \frac{\partial c}{\partial x} - B a_e E \frac{\partial }{\partial x} \left( N \frac{\partial c}{\partial x}\right) \right) \nonumber \\&-\mu \left( a_n N^2+B a_e NE \right) + \lambda c N(1-N-B E)^2, \end{aligned}$$

and

15 $$\begin{aligned} \frac{\partial E}{\partial t}= & {} \mu N\left( 1-N + 2 a_n N\right) -D (1-2 a_n) N \frac{\partial ^2 N}{\partial x^2} \nonumber \\&-\chi N\left( (1-a_n) \frac{\partial c}{\partial x} \frac{\partial N }{\partial x} + a_n \frac{\partial }{\partial x} \left( N \frac{\partial c}{\partial x}\right) \right) ,\nonumber \\ \end{aligned}$$

In Eqs. (14) and (15), the diffusion and chemotactic coefficients, *D* and $\chi$, are defined by

16 $$\begin{aligned} D = \frac{\mu h^2}{4} = \lim _{\tau \rightarrow 0} \frac{P_m h^2}{4 \tau }, \quad \chi = \mu k h^2 = \lim _{\tau \rightarrow 0} \frac{P_m k h^2}{\tau }, \end{aligned}$$

and $\mu$ and $\lambda$ are defined as

17 $$\begin{aligned} \mu = \lim _{\tau \rightarrow 0} \frac{P_m}{\tau }, \quad \lambda = \lim _{\tau \rightarrow 0}\frac{P_p}{\tau }. \end{aligned}$$

We remark that the expressions for *D*, $\chi$ and $\lambda$ are consistent with those derived in existing literature (Pillay et al. 2017; Simpson et al. 2009a, 2010; Codling et al. 2008), although we have not taken the limit $\tau , h \rightarrow 0$ with $h^2/\tau$ held constant (see Pillay et al. 2017). Furthermore, as we use small, non-zero, finite values for *h* and $\tau$ in the CA model simulations, and in the definitions of the continuum model parameters, retaining the $O(h^2)$ terms and neglecting higher-terms (used in Davies et al. 2014; Ross et al. 2015; Hywood et al. 2013) is reasonable.

As in Pillay et al. (2017), we use the column-averaged CA simulation results for the TCs and ECs at some later discrete time step $\bar{K}_{IC}>0$, $N_{i}(\bar{K}_{IC})$ and $E_{i}(\bar{K}_{IC})\, \forall i$ [ensemble averages defined in Eqs. (5 and (6)], respectively, as the initial conditions in the continuum model as follows:

18 $$\begin{aligned} N(x, t_{IC})\; \forall x,= & {} N_{i}(\bar{K}_{IC}) \; \forall i, \end{aligned}$$

19 $$\begin{aligned} E(x, t_{IC}) \; \forall x,= & {} E_{i}(\bar{K}_{IC}) \; \forall i, \end{aligned}$$

where $t_{IC}=\tau \bar{K}_{IC}$. In so doing, we avoid using the discontinuous TC initial condition [see Eq. (3)] in the discrete model at $\bar{K}=0$ to initialize the PDE model.

We approximate the boundary conditions by imposing no flux boundary conditions on Eq. (14), assuming only TC motility incorporating TC volume exclusion with probability $1-a_n$ as follows

20 $$\begin{aligned} D \frac{\partial N}{\partial x} - \chi N(1-(1-a_n)N) \frac{\partial c}{\partial x}\mid _{x=0,1} = 0. \end{aligned}$$

In other words, we assume that branching and TC annihilation via anastomosis are negligible at the boundaries. Given that we use initial conditions from the discrete model at a later time point once TCs have moved away from the boundary at $x=0$, and secondly, in the discrete model, TC movement at the TAF source is restricted, and no branching or anastomosis occurs at $x=1$, no flux boundary conditions are a reasonable approximation. Equations (14)–(20) define a one-dimensional model of angiogenesis that accounts for motility, anastomosis and branching.

## Structure of continuum models

We first consider Model 1 (see Table 1) by setting $B=0$ in Eq. (14). When $a_n=0$, the motility terms for the TC density reduce to well-known results for biased motility incorporating TC volume exclusion; the diffusion term is linear, while the chemotactic term is non-linear (Simpson et al. 2009a, 2010; Penington et al. 2011; Liggett 1999) as follows:

21 $$\begin{aligned} \frac{\partial N}{\partial t} = D \frac{\partial ^2 N}{\partial x^2} -\chi \frac{\partial }{\partial x} \left( N(1-N) \frac{\partial c}{\partial x}\right) + \lambda c N(1-N)^2. \end{aligned}$$

The chemotactic term can be written as $-\chi (1-2N)\frac{\partial N}{\partial x}$, since $\frac{\partial c}{\partial x} =1$ and $\frac{\partial ^2 c}{\partial x^2}=0$. We note that the resulting PDE for *N*(*x*, *t*) resembles a viscous Burgers’ equation (excluding the source term).

When $a_n = 1$, Eqs. (14) and (15) reduce to the non-volume excluding model derived in Pillay et al. (2017). In this case, tip-to-tip anastomosis gives rise to the usual non-linear sink term $-\mu N^2$, and leads to pre-multiplication of the diffusion and chemotactic terms by $1-N$ as follows:

22 $$\begin{aligned} \frac{\partial N}{\partial t} = D ( 1- N) \frac{\partial ^2 N}{\partial x^2} -\chi (1-N) \frac{\partial }{\partial x} \left( N\frac{\partial c}{\partial x}\right) -\mu N^2+\lambda c N(1-N)^2.\qquad \end{aligned}$$

When $a_n \in (0,1)$ our equation for the TC density is a combination of the diffusive and chemotactic terms that represent TC volume exclusion and tip-to-tip anastomosis [see Eq. (14)]. In Model 1, the non-linear branching term $\lambda c N (1-N)^2$ incorporates volume exclusion from TCs (and is consistent with a result derived previously by Simpson et al. 2010).

In Model 2, tip-to-sprout anastomosis gives rise to the term $-a_e E$ that pre-multiplies the diffusive and chemotactic terms, and a sink term $-\mu NE$. Thus, we recover the tip-to-sprout anastomosis terms derived in Pillay et al. (2017). The branching term in Model 2, $\lambda c N (1-N-E)^2$, also accounts for EC volume exclusion.

ECs are created via TC movement and tip-to-tip anastomosis. In Eq. (15), restated below:

$$\begin{aligned} \frac{\partial E}{\partial t}= & {} \mu N\left( 1-N + 2 a_n N\right) -D (1-2 a_n) N \frac{\partial ^2 N}{\partial x^2} \\&-\chi N\left( (1-a_n) \frac{\partial c}{\partial x} \frac{\partial N }{\partial x} + a_n \frac{\partial }{\partial x} \left( N \frac{\partial c}{\partial x}\right) \right) , \end{aligned}$$

$\mu$ can be interpreted as the TC motility rate, and, in effect, the source term $\mu N (1-N +2 a_n N)$ is a proliferation term. Tip-to-tip anastomosis also creates ECs, and this process is represented by the positive counterparts of the tip-to-tip anastomosis terms that appear in Eq. (14), as found in Pillay et al. (2017). The source term incorporates TC volume exclusion through the $1-N$ term, and there are additional negative flux terms that account for TC volume exclusion [see terms multiplied by $1-a_n$ in Eq. (14)]. Fewer ECs are produced when TC volume exclusion is active, as opposed to a non-volume exclusion model (see Pillay et al. 2017) where EC evolution is described entirely by $\mu N$. Given the form of the equations for *E*(*x*, *t*), we need only specify an initial condition for *E*.

Density restrictions are imposed through volume exclusion and anastomosis. In Model 1, $0\le N \le 1$ and in Model 2, $0\le N+E\le 1$ (see Table 1). With appropriate initial conditions, all models remain well-posed (provided we set $\lambda =0$ once maximum density has been reached i.e. $N=1$ in Model 1 and $N+E=1$ in Model 2, so that branching ceases). Thus, in contrast to the non-volume excluding model in Pillay et al. (2017), in Model 2, we can also account for tip-to-sprout anastomosis, while neglecting tip-to-tip anastomosis ($B=1, a_n = 0, a_e = 1$). We note that population behavior produced by averaged CA simulation results will always respect density restrictions, as occupancy restrictions can never be violated in the CA model.

## Mean-field approximation and behavior of PDE models

To compare the discrete and continuum models, we set micro- and macroscale parameters, related through Eqs. (16) and (17). Values of six (of the nine) independent microscale parameters, $R, h, P_m, \tau , k, K_{IC}$ are specified in Table 2, while $P_p$ (branching probability), $a_n$ (Model 1 and 2) and $a_e$ (Model 2 only), are fixed for each CA model case we consider. The movement probabilities are calculated using Eqs. (1) and (2), and also specified in Table 2.

**Table 2:** Summary of dimensionless values of microscale and macroscale model parameters

CA parameters	*R*	*h*	$P_m$	*k*	$\tau$	$K_{IC}$	$P^{x\pm }_{i,j}$	$P^{y\pm }_{i,j}$
	200	1/200	1	100	1/160	32	$\frac{1}{2}$, 0	$\frac{1}{4}$

PDE parameters	*D*	$\chi$	$\mu$	$t_{IC}$				
	$10^{-3}$	0.4	160	0.2				

$P^{x\pm }_{i,j}$ and $P^{y\pm }_{i,j}$ are calculated using Eqs. (1) and (2)

**Fig. 4:**
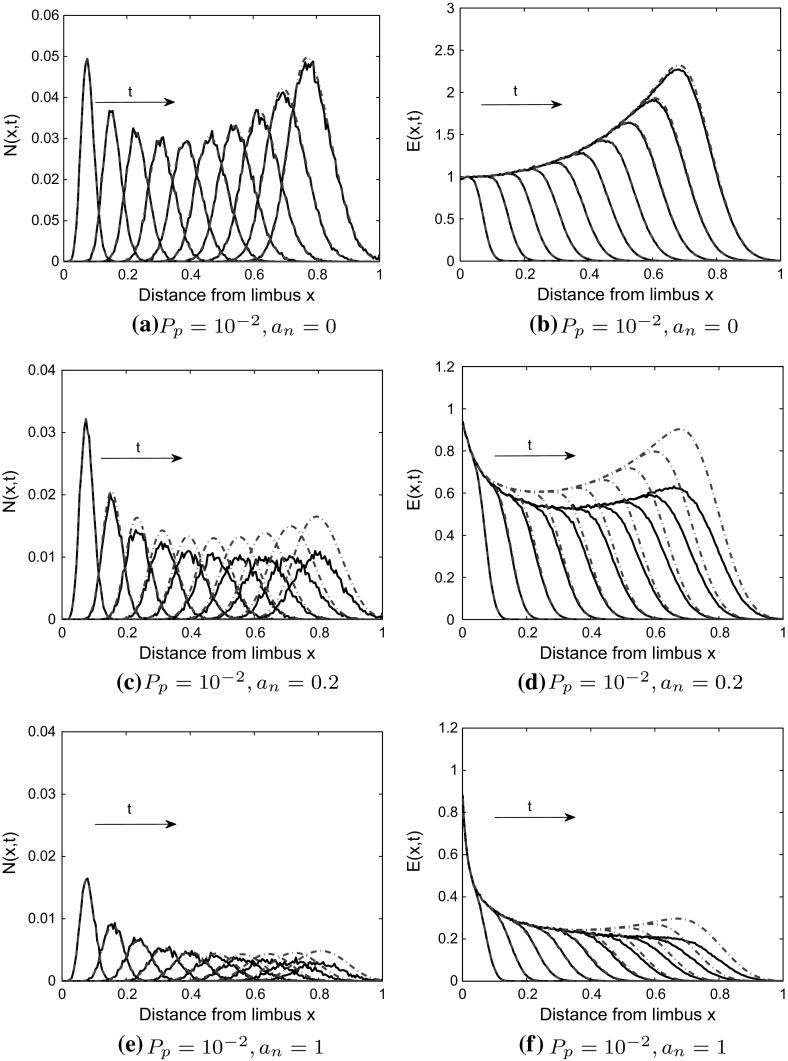
Model 1: effect of branching and anastomosis on agreement between PDE solutions and averaged CA simulation results. (Left panel) Tip cell (TC) density, (Right panel) Endothelial cell (EC) density. In the CA model, tip-to-tip anastomosis occurs with probability $a_n$, TC volume exclusion with probability $1-a_n$ and branching with probability $P_p$. **a**, **b** When $a_n=0$, there is good agreement between the PDE solutions (blue dashed-dot curves), Eqs. (14) and (15) with $B=0$, and averaged CA simulation results (black solid curves). **c**–**f** If $a_n=0.2, 1$, the agreement is poor, likely due to a break down of the mean-field approximation. Averaged CA simulation results and PDE solutions are shown at times $t=0.2, 0.4, \ldots , 2.0$ (color figure online)

The macroscale parameters $D, \chi , \mu , t_{IC}$ can be calculated directly [using Eqs. (16) and (17)] from the microscale parameters; their values are stated in Table 2. The parameter $\lambda$ can be calculated, using Eq. (17), once $P_p$ is set. The variables $a_n$ and $a_e$ also appear in the macroscale description, and may vary between CA model cases. A discussion justifying the choice of values for the microscale parameters, and the corresponding dimensional macroscale parameters, can be found in Pillay et al. (2017).

We now solve our PDE models and compare their outputs to column-averaged discrete simulation results, averaged over $M=200$ realizations. In Appendix B, we detail the methodology used to solve the PDEs.

**Fig. 5:**
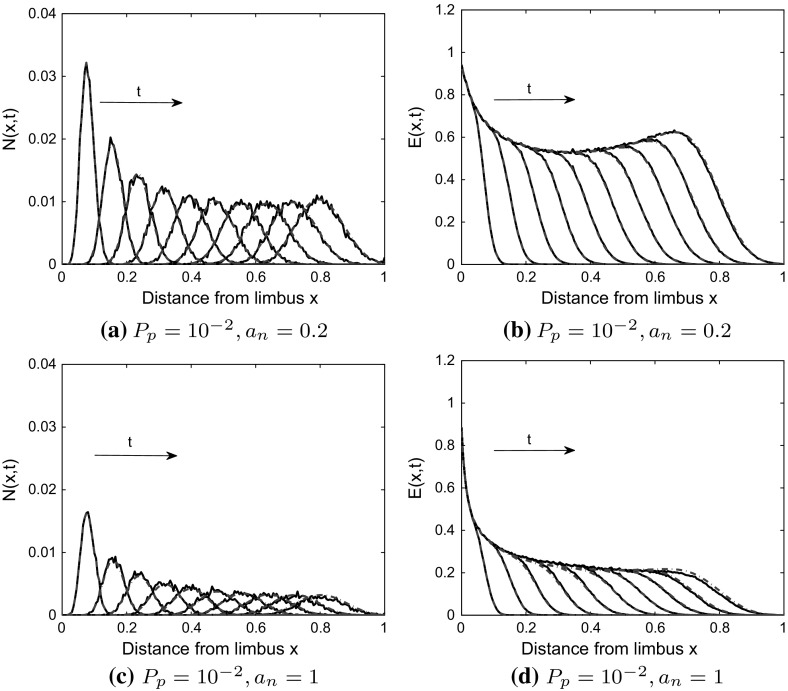
Model 1: agreement improved by fitting the PDE models to averaged CA simulation results. (Left panel) Tip cell (TC) density, (Right panel) Endothelial cell (EC) density. In the CA model, tip-to-tip anastomosis occurs with probability $a_n$, TC volume exclusion with probability $1-a_n$ and branching with probability $P_p$. **a**, **b** For $a_n = 0.2$, we estimate both $\tilde{\lambda }$ and $\tilde{a}_n$, and the PDE model solutions agree well with the averaged CA simulation results when $\tilde{\lambda } =1.1821\pm 0.0041$ and $\tilde{a}_n = 0.3001\pm 0.0008$. **c**, **d** There is good agreement between the PDE model solutions (blue dashed-dot curves) and discrete model simulations (black solid curves) when $\tilde{\lambda } =0.8582\pm 0.0083$. Averaged CA simulation results and PDE solutions are shown at times $t=0.2, 0.4, \ldots , 2.0$ (color figure online)

### Model 1

When $P_p=10^{-3}$ and $a_n=0, 1$, there is good agreement between the PDE solutions and averaged CA simulation results (see Fig. 12 in Appendix C). When the branching probability is increased from $P_p=10^{-3}$ to $P_p=10^{-2}$, we find agreement when $a_n = 0$ (see Fig. 4). However, for $a_n = 1$ and $a_n=0.2$, the agreement is poor (see Fig. 4). We postulate, therefore, that it is the increase in the branching probability to $P_p = 10^{-2}$, relative to the motility probability $P_m=1$, along with tip-to-tip anastomosis interactions, that leads to poor agreement between the PDE model and the CA simulation results (see Fig. 4c–f, we note that the agreement is poorer for $a_n=0.2$ than for $a_n=1$, possibly due to the way in which we have modeled anastomosis in the difference equations). We explain these results as follows: The PDEs were derived using a mean-field approximation which places constraints on the interactions that can occur in the CA, and their probabilities. For example, the branching probability, $P_p$, should be small compared to the motility probability, $P_m$, for the mean-field approximation to hold (Simpson et al. 2010; Baker and Simpson 2010; Davies et al. 2014). Further, the mean-field approximation may break down due to anastomosis, as the chances of an anastomosis event increases when agents are in close proximity to each other (Pillay et al. 2017). Other studies (Simpson and Baker 2011; Baker and Simpson 2010) have shown that high birth (branching) and death (anastomosis) rates (relative to the motility rate) enhance correlations between lattice sites. Thus, we postulate that it is a break down in the mean-field assumption that causes the discrepancy between our PDE solutions and averaged CA simulation results. Since it is intractable to correct our mean-field PDEs by accounting for correlations (see Baker and Simpson 2010; Markham et al. 2014; Simpson and Baker 2011) in our difference equation framework, we instead use the structure of the PDEs generated from the mean-field approximation and estimate parameters in the PDE model (by fitting the PDE models to the averaged CA simulation results) to account for the complexity of interactions. That is, we estimate through fitting those parameters that arise from processes that lead to correlations, namely $\lambda$ and $a_n$, the branching rate and parameter controlling tip-to-tip anastomosis, respectively. For clarity, we indicate by $\tilde{\lambda }$ and $\tilde{a}_n$ the continuum parameters that have been estimated by fitting the PDE models to averaged CA results.

The results presented in Fig. 5 reveal that when we estimate parameters in the continuum model, good agreement between the solutions of the PDE model and the CA simulation results can be found. When $a_n = 0$ (minimum value) or $a_n = 1$ (maximum value), we estimate only $\tilde{\lambda }$ in the continuum model. When $0< a_n < 1$, both $\tilde{a}_n$ and $\tilde{\lambda }$ must be estimated in the continuum model to find good agreement. (Details of the fitting procedure can be found in Appendix B.)

### Model 2

Following Pillay et al. (2017), we estimate the parameter controlling tip-to-sprout anastomosis, $a_e$, now renamed $\tilde{a}_e$, by fitting the PDE model to averaged CA simulation results. As mentioned in the PDE derivation, this enables us to account for prohibited self-loops, which are not explicitly accounted for in the difference equations. For $P_p = 10^{-3}$, $a_n = 1$ or 0, and $a_e = 1$ in the CA model, the PDE model solutions agree well with the averaged CA simulations when we estimate $\tilde{a}_e$ (see Fig. 13 in Appendix C).

Thus far, we have not derived a PDE model for which $a_e < 1$ in the CA model. In the CA model, as $a_e$ is decreased below 1, the probability of aborted TC moves due to EC volume exclusion, $1-a_e$, increases. This leads to a multi-species exclusion process (exclusion from TCs and ECs). Mean-field derivations of PDE models accounting for multi-species exclusion processes lead to highly non-linear advective-diffusion equations (see Simpson et al. 2009a). From the previous discussion on the break-down of the mean-field, it is likely that introducing further interactions (EC volume exclusion) will yield continuum models that cannot capture averaged CA simulation results. As we are already estimating the parameter $\tilde{a}_e$ by fitting the PDE model to averaged CA simulation results, we will also attempt to capture averaged CA simulation results generated with $0 < a_e \le 1$ through this methodology. Thus, we account for EC volume exclusion through parameter estimation, instead of using the difference equation framework to derive more detailed non-linear PDEs.

Setting $a_e=0$ in the CA corresponds to total EC volume exclusion and neglect of tip-to-sprout anastomosis. In reality, this is unlikely, as networks produced via angiogenesis show connections between sprouts. Therefore, we consider averaged CA simulations for $a_n = 1, 0$ and $a_e \in (0,1]$ in Model 2. To reproduce the brush-border effect (see Sect. 1), we use a high branching probability of $P_p = 10^{-1}$ in the CA model. Guided by the discussion on potential mean-field break-down in Model 1, in Model 2, we estimate $\tilde{\lambda }$ in addition to $\tilde{a}_e$, as we have increased the branching probability. We expect there will be a threshold value of $a_e$ in the CA model below which the Model 2 PDEs fail to capture the population behavior due to the increasing effects of EC volume exclusion.

In Sect. 6, we compare solutions to Eqs.  (14) and (15) with parameters estimated through fitting to column-averaged CA simulation results to assess agreement.

**Table 3:** Cases for CA and PDE models: we give the CA model parameters, and the corresponding parameters in the continuum model

CA model	$a_n$	$a_e$	$P_p$
1	[0, 1]	N/A	$10^{-2}, 4\times 10^{-2}$
2	0 or 1	(0, 1]	$10^{-1}$

PDE model	$\tilde{a}_n$	$\tilde{a}_e$	$\tilde{\lambda }$
1 [$B=0$ in Eq. (14)]	[0,1]	N/A	[0, 1.6], [0, 6.4]
2 [$B=1$ in Eq. (14)]	0 or 1	(0, 1]	[0, 16]

In the PDE models, the parameters are estimated by fitting the PDE models to averaged CA simulation results, with the exception of $\tilde{a}_n$ in Model 2, which is the direct analogue of $a_n$ in the CA model

### BC model

We will also compare (in Sect. 6) the modified BC model (see Appendix A) to the averaged CA simulation results. We estimate the BC model parameters ($D_{bc}, \chi _{bc}, \lambda _{bc}, \beta _e$ and $\beta _n$) by fitting the model to averaged CA simulation results. In so doing, we determine whether non-linear interactions and volume exclusion can be accounted for through the parameters in a linear model (linear in *N*).

## Discrete-continuum comparisons in one dimension with parameters estimated through fitting

In Table 3, we outline the CA (see Table 1) and PDE model cases we will compare (see Table 2 for values of other model parameters). The fitted parameter values for the PDE models are stated in Appendix D (see Tables 4, 5, 6, 7). For Models 1 and 2, we have used high branching probabilities (relative to the motility probability $P_m=1$) to ensure that the TC density increases as the TC front approaches the TAF source and thereby qualitatively reproduce the brush border effect (see Sect. 1).

We will compare the solutions of the one-dimensional Eqs. (14)–(20) and the BC model [Eqs. (23) and (24)] to averaged CA simulation results [see Eqs.  (5) and (6)], where lattice site occupancy has been averaged over $M=200$ realizations and $R=200$ columns. We also present simulation results from individual realizations of the CA model to illustrate the effects of volume exclusion on network morphology.

**Fig. 6:**
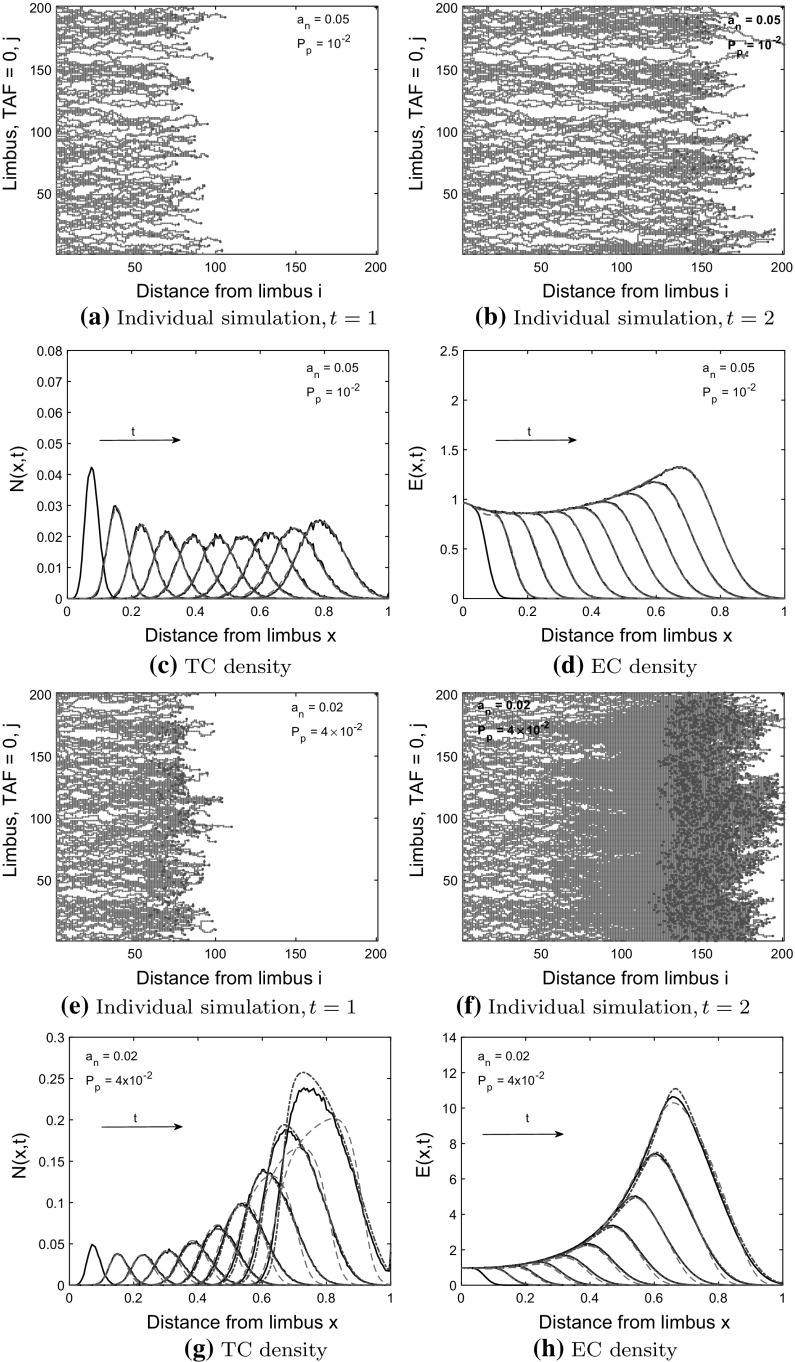
Model 1: comparisons between PDE solutions and averaged CA simulation results. TC and EC densities increase as the probability of tip-to-tip anastomosis, $a_n$, decreases and the branching probability, $P_p$ increases. **a**, **b**, **e**, **f** Individual simulations from the CA shown at $t=1$ and $t=2$, red dots $=$ ECs, light blue circles $=$ TCs. Forward TC movement is prevented by TC volume exclusion, resulting in a wider TC front in (**f**) than in (**b**). **c**, **d**, **g**, **h** Column-averaged CA simulation results (black solid curves) are shown at times $t=0.2, 0.4, \ldots , 2.0$ (arrow shows direction of increasing time). The TC front steepens at the back (see **g**), as TC volume exclusion prevents the forward movement of TCs as the TC density increases. The CA simulation results at $t_{IC} = 0.2$ are used as the initial conditions in the Model 1 PDEs (blue dashed-dot curves) and the BC model (red dashed curve) with the PDE solutions shown from $t = 0.4$ to $t = 2.0$ (color figure online)

### Model 1 Comparisons

**Fig. 7:**
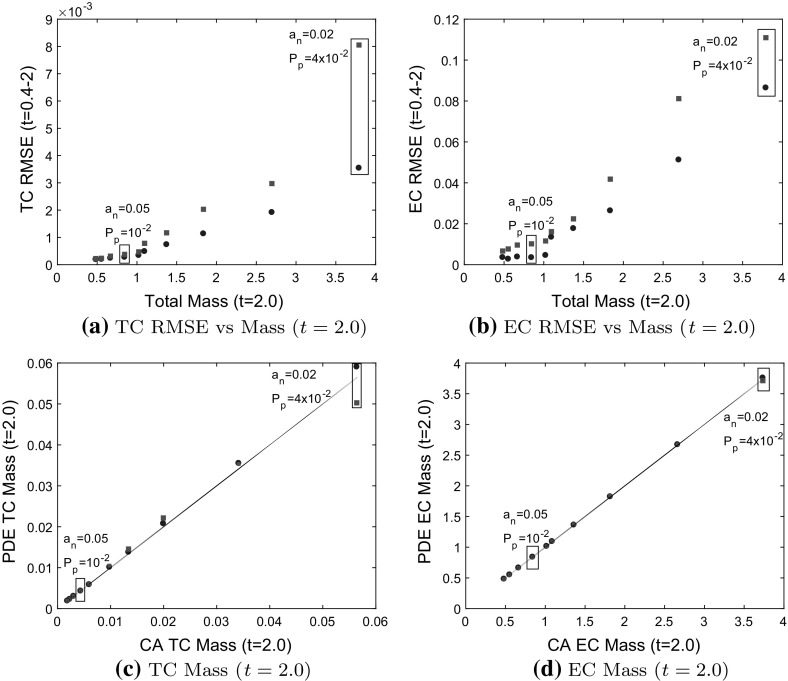
Model 1: comparisons between PDE solutions and averaged CA simulation results. TC and EC densities increase as the probability of tip-to-tip anastomosis, $a_n$, decreases and the branching probability, $P_p$ increases. **a**, **b** The relative difference between the root mean square error (RMSE) for each model increases as mass (integral of cell densities over *x*) at $t=2.0$ increases. **c**, **d** Mass from the PDE models at $t=2.0$ agrees with mass from the CA model (black line), except when TC volume exclusion takes effect at high TC density. The PDE models agree with the CA simulation results when the total cell density/mass is low, but the BC model deviates from the CA simulation results as the cell density increases, seen in (**a**), (**c**) and Fig. 6g. Boxed data points correspond to annotated parameter values, Model 1 PDEs $=$ blue circles, BC Model $=$ red squares (color figure online)

In Fig. 7, we compare Model 1 PDEs and the BC model to averaged CA simulation results for which tip-to-tip anastomosis occurs with probability $a_n$, and TC volume exclusion with probability $1-a_n$. We use a branching probability of $P_p=10^{-2}$ and $P_p = 4\times 10^{-2}$ for each of $a_n=0.02, 0.05, 0.1, 0.15, 0.2$. We see that as the probability of tip-to-tip anastomosis decreases, and the branching probability increases, the TC and EC densities at later time points increase due to reduced TC death rate and increased TC birth rate (compare Fig. 6c, d, g, h). The larger population of TCs causes the TC density (see Fig. 6f–h) to steepen at the back and extend to the left as forward TC movement is prevented by TC volume exclusion (contrast this behavior with that depicted in Fig. 6b–d). Steepening of the TC front can be understood by considering the PDE for the TC density. When $a_n=0$, a factor of $1-2N$ pre-multiplies the chemotactic term $\frac{\partial N}{\partial x}$ in Eq. (14). We can approximate the wave speed as $1-2N$, which decreases as *N* increases, causing the TC front to steepen at the back. This is analogous to the steepening behavior that occurs in Burgers’ equation (see Whitham 1974). Thus, as we reduce $a_n$ in our CA model, if the cell density is high, a front that steepens at the back will develop. A model that is linear in the chemotactic term will not generate this behavior. Thus, the BC model, in which the chemotactic term ($\frac{\partial N}{\partial x}$) is linear in *N* cannot capture this steepening behavior and, therefore, deviates from the averaged CA simulation results (see Fig. 6g).

**Fig. 8:**
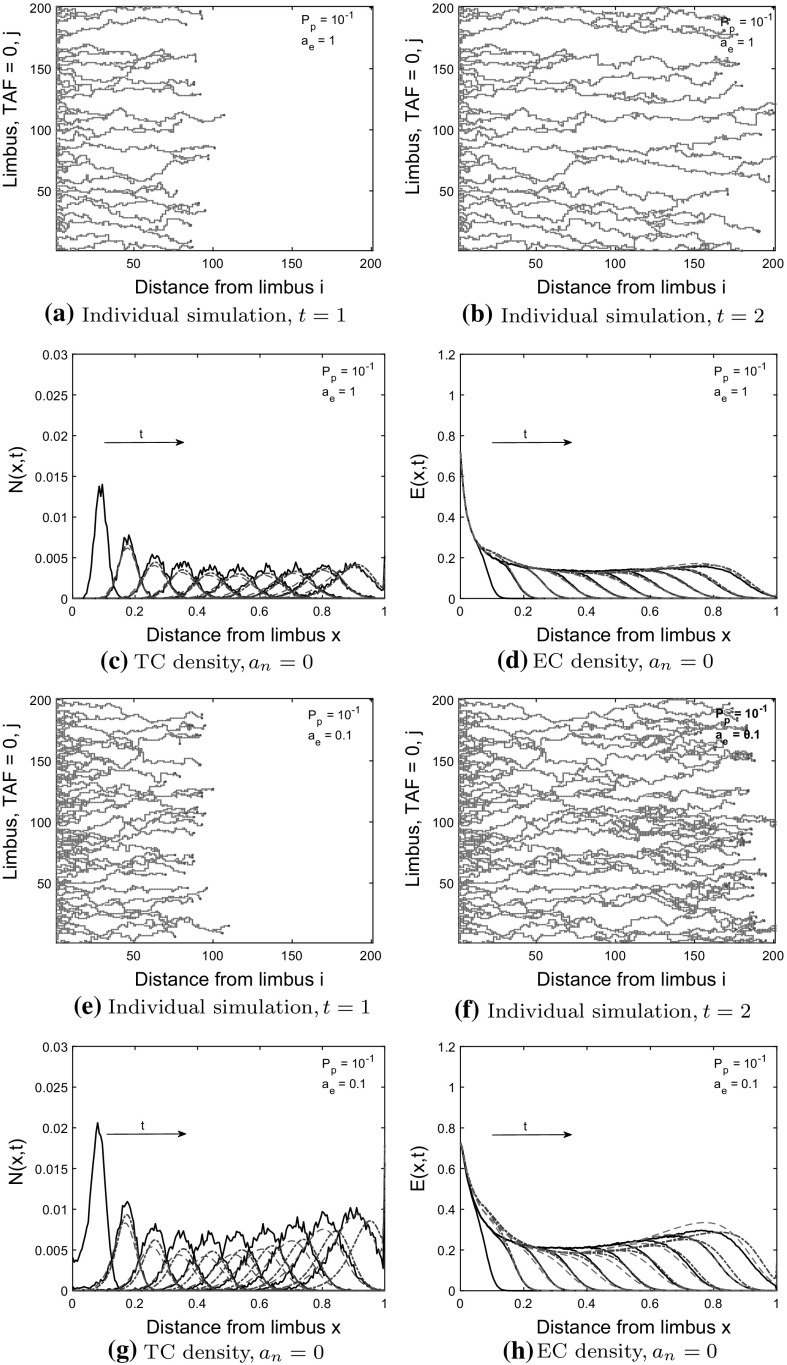
Model 2 ($$a_n=0)$$: comparisons between PDE solutions and averaged CA simulation results. TCs interact with ECs through tip-to-sprout anastomosis and EC volume exclusion, and with TCs through TC volume exclusion. TC and EC densities increase as the probability of tip-to-sprout anastomosis, $$a_e$$, decreases. The branching probability is $$P_p=10^{-1}$$. **a**, **b**, **e**, **f** Individual simulations from the CA shown at $$t=1$$ and $$t=2$$, red dots $$=$$ ECs, light blue circles $$=$$ TCs. The networks are sparser than in Fig. 6a, b, e, f due to tip-to-sprout anastomosis and EC volume exclusion. **c**, **d**, **g**, **h** Column-averaged CA simulation results (black solid curve) are shown at times $$t=0.2, 0.4, \ldots , 2.0$$ (arrow shows direction of increasing time). The migrating TC front exhibits long tails (see **g**), as EC volume exclusion prevents movement of TCs as the EC density increases. The CA simulation results at $$t_{IC} = 0.2$$ are used as the initial conditions in the derived PDE model (blue dashed-dot curve) and the BC model (red dashed curve), with the PDE solutions shown from $$t = 0.4$$ to $$t = 2.0$$ (color figure online)

The root mean square error (RMSE, see Eqs. (26) and (27) in Appendix B), provides a measure of the mismatch between the PDE solutions and the averaged CA results. The RMSE increases for both models as the total cell mass (integral of cell densities over *x* at $t=2.0$) increases (see Fig. 7a, b). Therefore, the relative difference between the RMSE for each model should be used to determine which model is a better fit (lower RMSE). When the mass is low and TC volume exclusion effects are negligible, both the Model 1 PDEs and the BC model agree well with the averaged CA simulation results, and thus the RMSEs for both models are clustered together at low values. As the TC density increases, the BC model deviates from the averaged CA simulation results (see Fig. 6g where TC volume exclusion effects are visible) and the relative difference between the RMSEs increases. Both models capture total cell mass at $t=2.0$ well except when TC volume exclusion effects are noticeable (see Fig. 7c, d). To summarize, as the TC density increases (for higher branching rates), the Model 1 PDEs better capture the non-linear behavior in the TC density that arises from TC volume exclusion than the BC model.

### Model 2 comparison

We compare the Model 2 PDEs and the modified BC model to averaged CA simulation results where tip-to-sprout anastomosis occurs with probability $a_e$, and EC volume exclusion with probability $1-a_e$. Recall that in this case branching incorporates two species exclusion. Additionally, any attempts at tip-to-sprout anastomosis within the same sprout are aborted incorporating an additional EC volume exclusion effect. In what follows, we consider two cases, one in which tip-to-tip anastomosis is neglected ($a_n=0$), and a second case in which tip-to-tip anastomosis occurs with probability 1 $(a_n=1)$. We use a branching probability of $P_p=10^{-1}$ for each of $a_e=0.1, 0.2, 1$ in the CA.

#### Case 1: TC volume exclusion occurs with probability 1 ($a_n =0$ in the CA model)

**Fig. 9:**
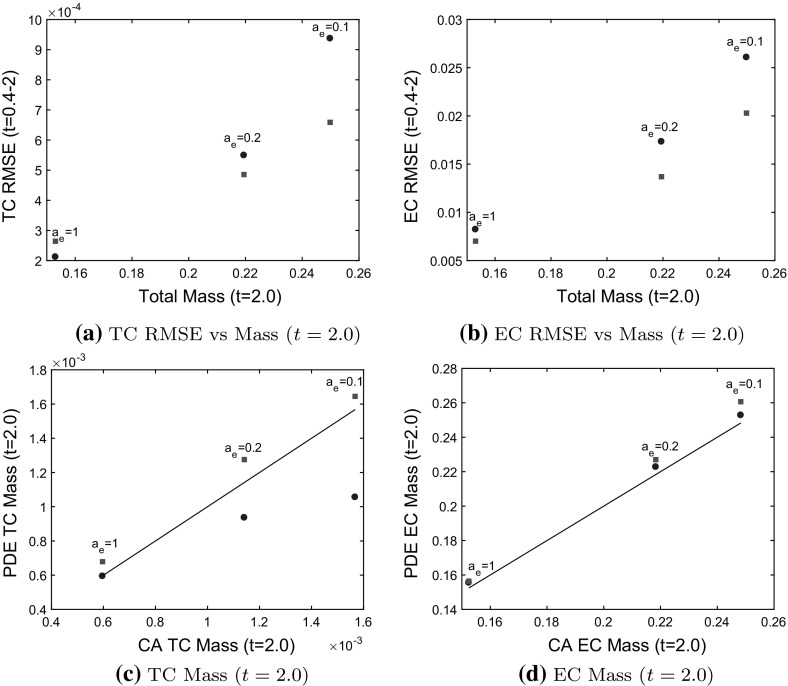
Model 2 ($$a_n=0)$$: comparisons between PDE solutions and averaged CA simulation results. TCs interact with ECs through tip-to-sprout anastomosis and EC volume exclusion, and with TCs through TC volume exclusion. TC and EC densities increase as the probability of tip-to-sprout anastomosis, $$a_e$$, decreases. The branching probability is $$P_p=10^{-1}$$. **a**–**d** Model 2 PDEs $$=$$ blue circles, BC Model $$=$$ red squares. **a**, **b** The relative difference between the root mean square error (RMSE) for each model increases as mass (integral of cell densities over *x*) at $$t=2.0$$ increases. **c**, **d** The Model 2 PDEs deviate from the CA TC mass (black line) at $$t=2.0$$ as stronger EC volume exclusion takes effect ($$a_e=0.1$$). The BC model agrees better with the CA simulation results for $$a_e=0.1$$ (color figure online)

As the probability of tip-to-sprout anastomosis, $a_e$, decreases, the cell densities/mass at $t=2.0$ (see Figs. 8, 9) increase as fewer TCs are annihilated via tip-sprout anastomosis. Thus, there are more TCs available to interact with ECs via volume exclusion. As TCs are excluded by ECs, long tails form in the TC density, as seen in Fig. 8g. This behavior contrasts with TC volume exclusion in Model 1, where forward movement of TCs is restricted by other TCs at the head of the migrating TC front.

The Model 2 PDEs and BC model are consistent with the averaged CA simulations when $a_e=1$ (see Figs. 8c, d, 9a–d). As $a_e$ decreases (EC volume exclusion more pronounced), the RMSE for the Model 2 PDEs increases more rapidly than for the BC model. The Model 2 PDEs produce a TC front that is out of phase with the averaged CA results at $t=2.0$ (see Fig. 8g for $a_n=0.1$; PDE solution is peaked ahead of the averaged CA simulation results at $t=2.0$). For $t < 2.0$, the Model 2 PDEs agree better with the averaged CA simulation results. In terms of EC mass at $t=2.0$, the Model 2 PDEs and the BC model agree well with the CA EC mass (see Fig. 9d). For $P_p = 10^{-1}, a_e = 1$ and $P_p = 10^{-2}, a_e=1$ (see Fig. 14 in Appendix C), both PDE models are in good agreement with the averaged CA simulation results. For $P_p = 10^{-1}, a_e = 0.2, 0.1$, the BC model captures the averaged CA simulation results better, according to the RMSE and TC mass metrics at $t=2.0$ (see Fig. 9a–d). For $P_p=10^{-2}, a_e = 0.2, 0.1$, the Model 2 PDEs capture the averaged CA simulation results better, according to the RMSE and TC mass metrics (see Fig. 14 in Appendix C). For values of $a_e$ below 0.1, as the effects of EC volume exclusion increase, neither model gives good agreement with the averaged CA simulation results (results not shown).

The TC density for Model 2 is much lower than the TC density for Model 1 (see Fig. 6, 7) for two reasons. Firstly, EC and TC volume exclusion in the branching process mean that fewer TCs are produced, even though the branching probability is higher than for Model 1. Secondly, TC-EC interactions annihilate many TCs via tip-to-sprout anastomosis. As the TC mass remains low, the TC volume exclusion effect seen in Model 1 (steepening of TC front) is not observed in Model 2.

#### Case 2: tip-to-tip anastomosis occurs with probability 1 ($a_n = 1$ in the CA model)

**Fig. 10:**
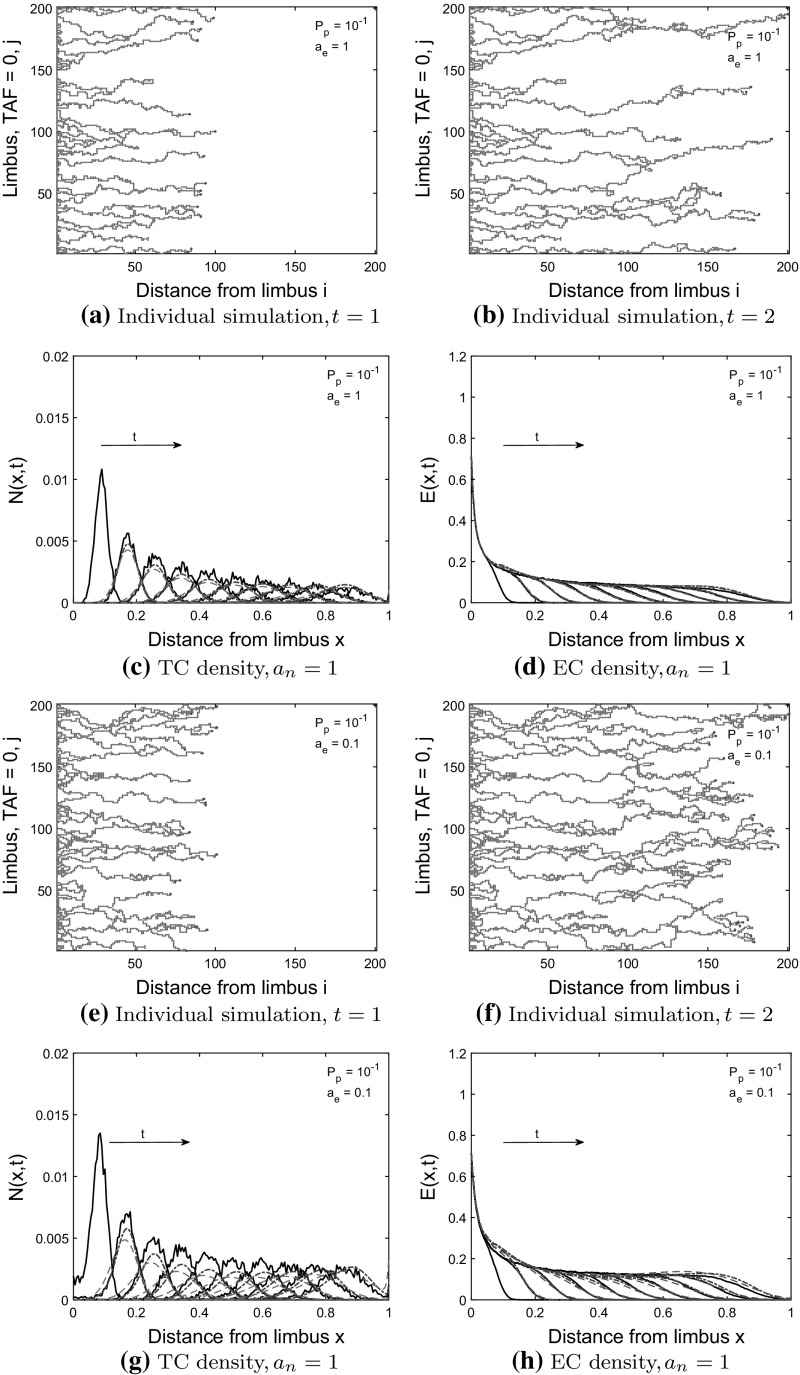
Model 2, TC volume exclusion neglected ($a_n=1$): Comparisons between PDE solutions and averaged CA simulation results. TCs interact with ECs through tip-to-sprout anastomosis and EC volume exclusion, and TCs are annihilated through tip-to-tip anastomosis. TC and EC densities increase as the probability of tip-to-sprout anastomosis, $a_e$, decreases. The branching probability is $P_p=10^{-1}$. **a**, **b**, **e**, **f** Individual simulations from the CA shown at $t=1$ and $t=2$, red dots $=$ ECs, light blue circles $=$ TCs. The networks are sparser than when $a_n=0$ (see Fig. 8a, b, e, f) due to tip-to-tip anastomosis. **c**, **d**, **g**, **h** Column-averaged CA simulation results (black solid curve) are shown at times $t=0.2, 0.4, \ldots , 2.0$ (arrow shows direction of increasing time). The migrating TC front exhibits short tails at early time points (t$=$0.4–0.8) (see **g**), as EC volume exclusion prevents movement of TCs. The CA simulation results at $t_{IC} = 0.2$ are used as the initial conditions for the Model 2 PDEs (blue dashed-dot curve), and the BC model (red dashed curve), with the PDE solutions shown from $t = 0.4$ to $t = 2.0$ (color figure online)

**Fig. 11:**
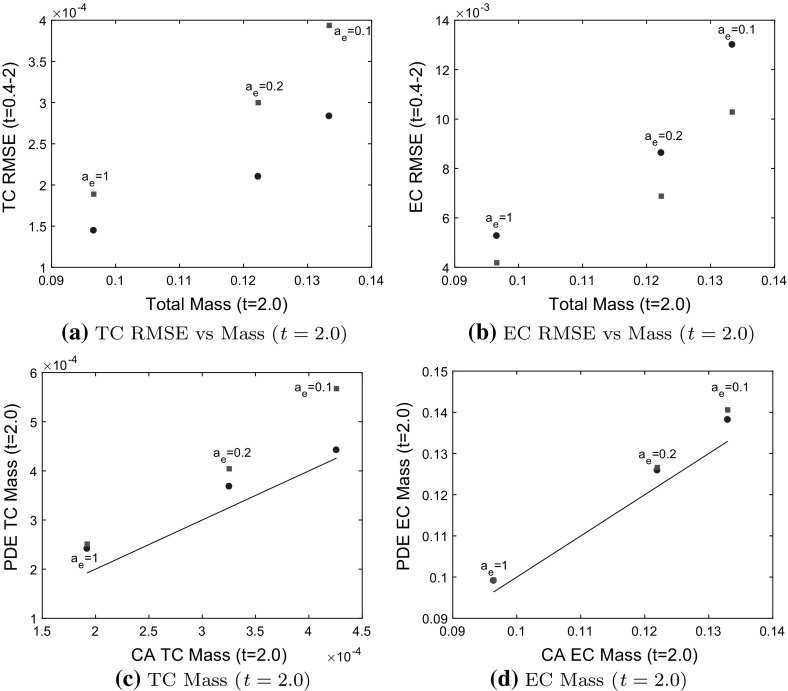
Model 2, TC volume exclusion neglected ($a_n=1$): Comparisons between PDE solutions and averaged CA simulation results. TCs interact with ECs through tip-to-sprout anastomosis and EC volume exclusion, and TCs are annihilated through tip-to-tip anastomosis. TC and EC densities increase as the probability of tip-to-sprout anastomosis, $a_e$, decreases. The branching probability is $P_p=10^{-1}$. **a**–**d** Model 2 PDEs $=$ blue circles, BC Model $=$ red squares. **a**, **b** The relative difference between the root mean square errors (RMSEs) are comparable for $a_e=0.2$ and $a_e=0.1$, but larger than for $a_e=1$. **c**, **d** The mass (integral of cell densities over *x*) calculated from both models are comparable to the CA mass (black line), though the TC mass for the BC model is slightly higher for $a_e=0.1$. Both models agree with the CA simulation results in this case, though the Model 2 PDEs perform better according to TC RMSE, TC and EC mass metrics (see **a**, **c**, **d**) (color figure online)

**Fig. 12:**
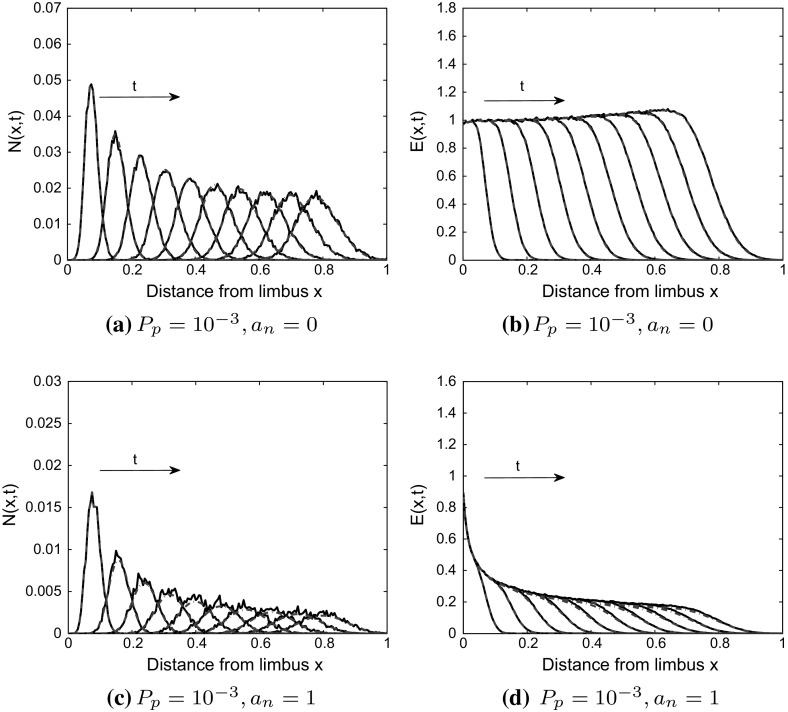
Model 1: effect of TC volume exclusion ($a_n=0$) and tip-to-tip anastomosis ($a_n=1$) on population behavior. (Left panel) TC density, (Right panel), EC density. There is good agreement between the PDE solutions (blue dashed-dot curves), Eqs. (14) and (15) with $B=0$, and averaged CA simulation results (black solid curves). Averaged CA simulation results and PDE solutions are shown at times $t=0.2, 0.4, \ldots , 2.0$ (color figure online)

**Fig. 13:**
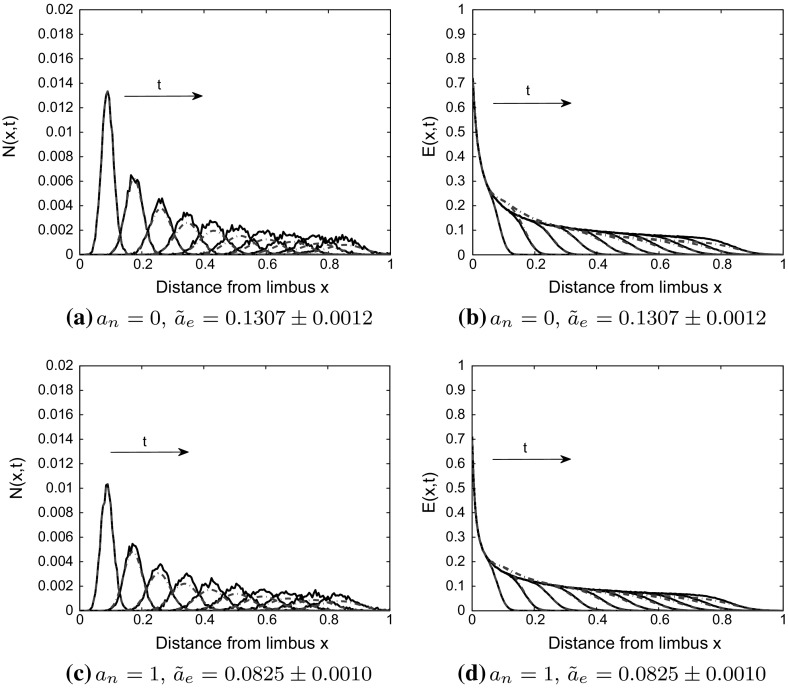
Model 2: effect of TC volume exclusion ($a_n=0$) and tip-to-tip anastomosis ($a_n=1$) on population behavior with branching probability $P_p=10^{-3}$. (Left panel) TC density, (Right panel) EC density. There is good agreement between the PDE solutions (blue dashed-dot curves), Eqs. (14) and (15) with $B=1$, and averaged CA simulation results (black solid curves). Averaged CA simulation results and PDE solutions are shown at times $t=0.2, 0.4, \ldots , 2.0$ over 400 runs (color figure online)

**Fig. 14:**
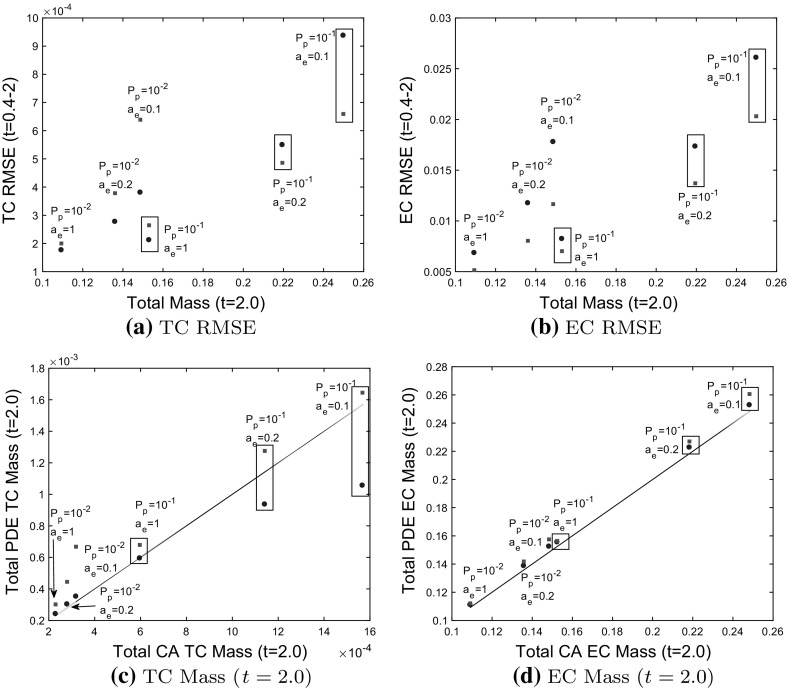
Model 2 ($a_n=0$): Model 2 PDEs $=$ blue circles, BC Model $=$ red squares. **a**, **b** For each value of $P_p$, the relative difference between the RMSEs for each model increases as $a_e$ decreases. Note that the RMSEs for each value of $P_p$ are not directly comparable, as RMSEs are not normalized with respect to mass. Instead, for each value of $P_p$, the difference between the RMSEs for the BC Model and Model 2 PDEs for each value $a_e$ indicates the goodness of fit. **c**, **d** The PDE models approximate the mass calculated from the CA model (black line) well, except for $P_p=10^{-1}, 10^{-2}$ and $a_e=0.1$. In these cases, for $P_p=10^{-1}$, the BC model approximates the TC mass better than the Model 2 PDEs (higher TC RMSE), while for $P_p=10^{-2}$, the Model 2 PDEs approximate the TC mass better (lower TC RMSE). Note that the goodness of fit becomes poorer as $a_e$ decreases and EC volume exclusion becomes more important. Boxed data: $P_p=10^{-1}$, Unboxed data: $P_p=10^{-2}$. Estimated parameter values are given in Tables 6 and 7 (color figure online)

**Fig. 15:**
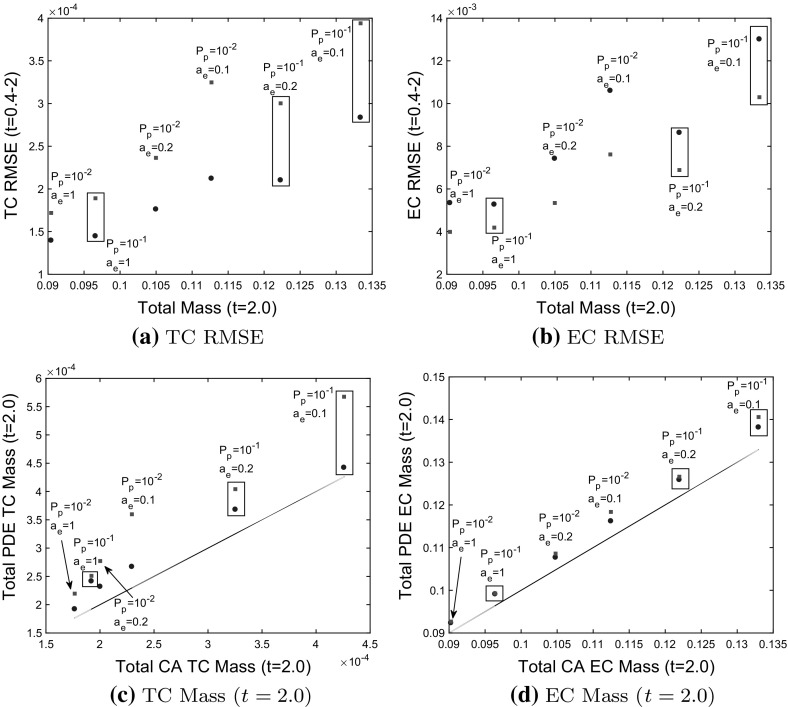
Model 2, TC volume exclusion neglected, ($a_n=1$): Model 2 PDEs $=$ blue circles, BC Model $=$ red squares. **a**, **b** TC and EC RMSEs. For each value of $P_p$, the difference between the RMSEs for the BC Model and Model 2 PDEs for each value $a_e$ indicates the goodness of fit. **c**, **d** All models overestimate the true CA mass (black line). Generally, both PDE models approximate the averaged CA simulation results well. Boxed data: $P_p=10^{-1}$, Unboxed data: $P_p=10^{-2}$. Estimated parameter values are given in Tables 6 and 7 (color figure online)

In Fig. 11, tip-to-tip anastomosis occurs with probability $a_n=1$, and TC volume exclusion is neglected. Both PDE models give good agreement with the averaged CA simulation results. For $a_e = 0.1$, EC volume exclusion produces short tails in TC density (see Fig. 10g). However, TC annihilation caused by tip-to-tip anastomosis means that EC volume exclusion effects are less pronounced than when tip-to-tip anastomosis is inactive (see Fig. 8g). We have also confirmed that both PDE models agree with the averaged CA simulation results for $P_p = 10^{-2}$ (see Fig. 15 in Appendix C). Based on the TC RMSE and TC mass metrics (see Fig. 11a, c, d), we conclude that the Model 2 PDEs agree slightly better with the averaged CA simulation results than the BC model. When $0< a_e < 0.1$, neither model captures the long tails in the TC density caused by EC volume exclusion (results not shown).

## Discussion and conclusion

We have developed two-dimensional CA models of corneal angiogenesis based on the snail-trail approach, explicitly accounting for cell volume. In Model 1, TCs interact with TCs via tip-to-tip anastomosis and TC volume exclusion. In Model 2, in addition to TC-TC interactions, TCs also interact with ECs through tip-to-sprout anastomosis and EC volume exclusion. We used a mean-field approximation to derive one-dimensional PDE models based on these CA models. We compared averaged CA simulation results with our PDE models and an existing phenomenological model (the BC model), which is linear in the diffusive, chemotactic and branching terms (assuming a linear TAF concentration), in order to determine macroscale volume exclusion effects and whether linear models can account for them.

We note that the PDE models are derived using a mean-field approximation, which places constraints on the probabilities with which interactions can occur in the CA model. It is well known that high birth (branching) and death (anastomosis) rates (relative to the motility rate) give rise to correlation effects that cannot be captured by mean-field continuum models. Thus, we postulate that deviations of our PDE model solutions from the averaged CA simulation results, which occur for high branching rates and/or active anastomosis, are caused by a break down in the mean-field assumption. We rectified this discrepancy by fitting the PDE models to averaged CA simulation results and estimating parameters in the PDE models that control processes from which correlations arise. In particular, we estimated the branching rate, and the parameters that control tip-to-tip anastomosis and tip-to-sprout anastomosis, in Model 1 and 2, respectively. We also used this approach to capture EC volume exclusion effects instead of deriving more complicated non-linear mean-field PDE models, which are unlikely to capture the averaged CA simulation results without estimating parameters through fitting.

We found that our Model 1 PDEs and the BC model were in good agreement with averaged CA simulation results when TC mass is low, and TC volume exclusion effects negligible. However, when the TC mass is high, averaged CA simulations indicate that TC volume exclusion produces a front that steepens at the back as TCs are prevented from moving towards the TAF source by other TCs. As the TC mass increases, the BC model is unable to capture the increasing effects of TC volume exclusion, while the non-linear chemotactic terms in the Model 1 PDEs have been derived for this purpose.

In Model 2, both the BC Model and the Model 2 PDEs capture the averaged CA simulation results when EC volume exclusion effects are negligible at the macroscale. As we reduce the probability of tip-to-sprout anastomosis in the CA model, thus increasing macroscale EC volume exclusion effects, long tails in the TC density form, as TCs are rendered immobile behind the migrating TC front (within the EC front). This behavior contrasts with TC volume exclusion effects, where forward movement of TCs is restricted by other TCs located ahead of the EC front (therefore, TCs are located within the TC front). Neither PDE model captures the increasingly long tails in the TC density due to EC volume exclusion. In the context of angiogenesis, however, network connections are prevalent, and thus, strong EC volume exclusion effects, which prevent connections via tip-to-sprout anastomosis, may not be relevant.

More generally, we note that TC-EC interactions in the CA and PDE models produce less dense networks than when TCs interact with TCs only, as tip-to-sprout anastomosis annihilates TCs, and EC volume exclusion further prohibits TC movement. Further, for vessel networks that are less dense, the pronounced effects of TC volume exclusion do not arise (as the TC density is low). This should be considered when developing angiogenesis models, as including or excluding TC-EC interactions has a marked effect on the vessel densities.

We note that by fitting the mean-field PDE models to averaged CA simulation results to estimate parameters, we are able to capture more complicated scenarios in the PDE and CA models. For example, we could implement branching and/or anastomosis when a sprout is of a certain length (i.e. length restrictions) in the CA model. Instead of deriving more complicated non-linear PDE models to capture these interactions, we exploit the structure of the mean-field PDEs and fit them to CA simulations to estimate the branching rate and parameters controlling anastomosis. This approach enables us to include a wider range of interactions in the CA model that would have been intractable to include and generalize in our discrete to continuum framework. We note, however, that a different approach is required to capture pronounced EC volume exclusion effects, and in future work, we intend to derive such a model. We will also consider how different discrete models, such as the cellular Potts model, affect the form of the PDEs derived. In future work, we aim also to determine the biological relevance of the parameter regimes in which our PDEs and the BC model agree/disagree with the averaged simulation results.

## Appendices

### A Review of Byrne and Chaplain’s snail-trail model 1995

We use the following modified version of Byrne and Chaplain’s dimensionless model of angiogenesis (see Byrne and Chaplain 1995; Spill et al. 2015; Pillay et al. 2017 for details):

23 $$\begin{aligned}&\frac{\partial N}{\partial t} = D_{BC} \frac{\partial ^2 N}{\partial x^2} - \chi _{BC} \frac{\partial }{\partial x} \left( N \frac{\partial c}{\partial x} \right) +\lambda _{BC} Nc - \beta _{e} NE - \beta _n N^2, \end{aligned}$$

24 $$\begin{aligned}&\frac{\partial E}{\partial t} = \frac{2}{h}\left| D_{BC} \frac{\partial N}{\partial x} -\chi _{BC} N \frac{\partial c}{\partial x} \right| , \end{aligned}$$

25 $$\begin{aligned}&D_{BC}\frac{\partial N}{\partial x} - \chi _{BC} N \frac{\partial c}{\partial x} \mid _{x=0,1}= 0. \end{aligned}$$

Here, *N*(*x*, *t*) and *E*(*x*, *t*) are the TC and EC densities, respectively, *c*(*x*) is the TAF concentration and *h* is the lattice spacing from the CA model. The TAF source is located at $x=0$ and the limbus at $x=1$ with $(x,t) \in [0,1]\times [0,\infty )$. We impose no flux boundary conditions [Eq. (25)], with initial conditions [Eqs. (18) and (19)] and linear TAF profile, $c(x)=x$, taken from the CA model.

### B Numerical schemes

#### B.1 CA algorithm

We outline the CA algorithm below.

While $$\bar{K} \le \bar{K}_{\mathrm {final}} (=320)$$

1. Choose $\bar{N}$ TCs independently at random with replacement Loop 1: For 1 to $\bar{N}$
  - Choose a random number, $\bar{T} \in [0,1]$
  - If $\bar{T}\le P_m$, then the TC attempts to move as follows:
  - A random number $\bar{S} \in [0,1]$ is chosen
  - The TC moves according to probabilities $P^{x\pm }, P^{y\pm }$
  - Model 1 and Model 2: Choose a random number ${{P_{tt} \in [0,1]}}$. If target site is occupied by TCs and $P_{tt} < a_n$, tip-to-tip anastomosis occurs, else TC move is aborted
  - Model 2: Choose a random number ${{P_{ts} \in [0,1]}}$. Else if target site is occupied by ECs and $P_{ts} < a_e$, tip-to-sprout anastomosis occurs (no self-loops), else TC move is aborted
  - Model 1 and 2: Else if target site is unoccupied, movement is allowed
  - If a TC move has occurred, an EC is left behind
  End Loop 1
2. Choose $\bar{N}$ TCs at random with replacement Loop 2: For 1 to $\bar{N}$
  - Choose a random number, $\bar{R}\in [0,1]$
  - If $\bar{R} \le P_b$, then branching occurs, provided neighboring sites are unoccupied by TCs (Model 1 and 2) or by ECs (Model 2)
  End Loop 2
3. Increment time step: $\bar{K} = \bar{K}+1$ End While Loop

#### B.2 PDE solvers

We solve Eqs. (14)–(20) and Eqs. (23)–(25) numerically using the method of lines with finite differences in space. The resulting time-dependent ordinary differential equations are solved using MATLAB’s ode15s solver, which implements adaptive time-stepping (Shampine and Reichelt 1997).

#### B.3 Model fitting

We fit the models to the averaged CA simulation results by implementing non-linear least squares using MATLAB’s lsqnonlin routine, which implements a trust-region-reflective algorithm (Coleman and Li 1994, 1996). In particular, we perform the fitting using 30 random start points generated using MATLAB’s optimization toolbox (createOptimProblem) and lsqnonlin. The random start points are generated within pre-defined bounds as follows: for Model 1 (Model 2) PDEs, $\tilde{\lambda } \in [0,30]$ and $\tilde{a}_n \in [0,1]$ ($\tilde{a}_e \in [0.08,1]$). For the BC model: $\tilde{D}_{BC} \in [10^{-4}, 10^{-2}]$, $\tilde{\chi }_{BC} \in [0.2, 0.5]$, $\tilde{\beta }_n \in [0, 80]$ and $\tilde{\beta }_e \in [0, 80]$. The confidence intervals are calculated using nlparci and the Jacobian output from lsqnonlin. We fit the models to averaged CA simulation results over $t\in [0.4,2]$ in intervals of 0.2 for both the TC and EC densities.

We measure the goodness of fit in terms of root mean square error, RMSE, which is defined as

26 $$\begin{aligned} \mathrm {TC \,RMSE}= & {} \sqrt{\frac{\sum _{i=1}^{P} (N^i_{PDE}-N^i_{CA})^2}{P}}, \end{aligned}$$

27 $$\begin{aligned} \mathrm {EC\, RMSE}= & {} \sqrt{\frac{\sum _{i=1}^P(E^i_{PDE}-E^i_{CA})^2}{P}}, \end{aligned}$$

where *P* is the total number of points (all lattice points for times $t=0.4-2.0$ in increments of $t=0.2$, $P = 1809$) and $N_{PDE}$ and $E_{PDE}$ and $N_{CA}$ and $E_{CA}$ are the PDE and CA model TC and EC densities, respectively.

### C Additional figures

Please see Figs. 12, 13, 14 and 15.

### D Tables of fitted parameter values

The estimated parameter values for Model 1 are given in Tables 4 and 5. The estimated parameter values for Model 2 are given in Tables 6 and 7.

**Table 4:** Model 1: fitted parameter values. The CA model parameters $a_n$ and the continuum analogue of the branching probability $P_p$, $\lambda$, are also given. A branching rate of 1.6 corresponds to $P_p=10^{-2}$ and 6.4 corresponds to $P_p=4\times 10^{-2}$

$\lambda$	$a_n$	$\tilde{\lambda }$	$\tilde{a}_n$
1.6	0.02	$1.4719\pm 0.0029$	$0.0333\pm 0.0003$
1.6	0.05	$1.3920\pm 0.0025$	$0.0811\pm 0.0003$
1.6	0.1	$1.3310\pm 0.0033$	$0.1606 \pm 0.0005$
1.6	0.15	$1.2080\pm 0.0028$	$0.2297\pm 0.0005$
1.6	0.2	$1.1821\pm 0.0041$	$0.3001\pm 0.0008$
6.4	0.02	$5.4722\pm 0.0219$	$0.0216\pm 0.0010$
6.4	0.05	$4.9973\pm 0.0200$	$0.0574\pm 0.0013$
6.4	0.1	$4.4291\pm 0.0150$	$0.1039\pm 0.0014$
6.4	0.15	$4.0862\pm 0.0127$	$0.1590\pm 0.0016$
6.4	0.2	$3.8473\pm 0.0111$	$0.2164\pm 0.0017$

**Table 5:** Model 1: BC model fitted parameter values. The CA model parameters $a_n$ and the continuum analogue of the branching probability $P_p$, $\lambda$ are also given. A branching rate of 1.6 corresponds to $P_p=10^{-2}$ and 6.4 corresponds to $P_p=4\times 10^{-2}$. The continuum analogue of the diffusion and chemotactic coefficient is $D=10^{-3}$ and $\chi =0.4$

$\lambda$	$a_n$	$\tilde{D}_{BC}$	$\tilde{\chi }_{BC}$	$\tilde{\lambda }_{BC}$	$\tilde{\beta }_n$
1.6	0.02	$0.0011\pm 0.0000$	$0.3885 \pm 0.0001$	$1.4698\pm 0.0085$	$5.7575\pm 0.1306$
1.6	0.05	$0.0011\pm 0.0000$	$0.3911 \pm 0.0001$	$1.3744\pm 0.0084$	$13.1802\pm 0.1498$
1.6	0.1	$0.0010\pm 0.0000$	$0.3926 \pm 0.0002$	$1.3265\pm 0.0094$	$25.9502\pm 0.2108$
1.6	0.15	$0.0010\pm 0.0000$	$0.3940 \pm 0.0002$	$1.2025\pm 0.0089$	$36.5733\pm 0.2291$
1.6	0.2	$0.0010\pm 0.0000$	$0.3936 \pm 0.0002$	$1.2000\pm 0.0088$	$48.0085 \pm 0.2634$
6.4	0.02	$0.0006 \pm 0.0000$	$0.3729 \pm 0.0003$	$8.7030 \pm 0.1141$	$31.1261 \pm 0.7475$
6.4	0.05	$0.0009 \pm 0.0000$	$0.3775 \pm 0.0003$	$6.4969\pm 0.0969$	$28.4414 \pm 0.9530$
6.4	0.1	$0.0012 \pm 0.0000$	$0.3833 \pm 0.0002$	$4.4815 \pm 0.0508$	$20.6232 \pm 0.7463$
6.4	0.15	$0.0012 \pm 0.0000$	$0.3877 \pm 0.0002$	$3.8890 \pm 0.0268$	$24.6209 \pm 0.4986$
6.4	0.2	$0.0011 \pm 0.0000$	$0.3905 \pm 0.0002$	$3.7604 \pm 0.0202$	$34.9179 \pm 0.4415$

**Table 6:** Model 2: fitted parameter values. The CA model parameters $a_e$ and the continuum analogue of the branching probability $P_p$, $\lambda$, are also given. A branching rate of 16 (1.6) corresponds to $P_p=10^{-1}$ ($P_p=10^{-2}$)

$\lambda$	$a_e$	$\tilde{\lambda }$	$\tilde{a}_e$
$a_n=0$	$\tilde{a}_n=0$		
1.6	0.1	$2.4145 \pm 0.0756$	$0.1520\pm 0.0019$
1.6	0.2	$2.0548 \pm 0.0560$	$0.1500 \pm 0.0015$
1.6	1	$1.7080 \pm 0.0408$	$0.1572\pm 0.0014$
16	0.1	$5.5277 \pm 0.0705$	$0.1178\pm 0.0016$
16	0.2	$4.9136 \pm 0.0528$	$0.1197 \pm 0.0013$
16	1	$3.6257 \pm 0.0346$	$0.1373 \pm 0.0012$
$a_n=1$	$\tilde{a}_n=1$		
1.6	0.1	$1.8305 \pm 0.0578$	$0.1168\pm 0.0017$
1.6	0.2	$1.4894 \pm 0.0449$	$0.1104 \pm 0.0014$
1.6	1	$1.1739 \pm 0.0384$	$0.1093 \pm 0.0014$
16	0.1	$2.7588 \pm 0.0581$	$0.1001\pm 0.0017$
16	0.2	$2.2644 \pm 0.0434$	$0.0949 \pm 0.0014$
16	1	$1.5406 \pm 0.0344$	$0.1034 \pm 0.0013$

**Table 7:** Model 2: BC Model Fitted Parameter Values. The CA model parameters $a_e$ and the continuum analogue of the branching probability $P_p$, $\lambda$ are also given. A branching rate of 16 (1.6) corresponds to $P_p=10^{-1}$ ($P_p=10^{-2}$). The continuum analogue of the diffusion and chemotactic coefficient is $D=10^{-3}$ and $\chi =0.4$

$a_n=0$	$\tilde{a}_n=0$				
$\lambda$	$a_e$	$\tilde{D}_{BC}$	$\tilde{\chi }_{BC}$	$\tilde{\lambda }_{BC}$	$\tilde{\beta }_e$
1.6	0.1	$0.0068\pm 0.0002$	$0.2921 \pm 0.0027$	$2.4866 \pm 0.0535$	$14.1592 \pm 0.2273$
1.6	0.2	$0.0029\pm 0.0001$	$0.3443 \pm 0.0020$	$2.2874 \pm 0.0416$	$18.6796 \pm 0.2261$
1.6	1	$0.0016 \pm 0.0001$	$0.3713\pm 0.0012$	$1.9170 \pm 0.0341$	$22.1619 \pm 0.1872$
16	0.1	$0.0017\pm 0.0001$	$0.3610 \pm 0.0022$	$5.4489 \pm 0.0680$	$18.4322 \pm 0.2562$
16	0.2	$0.0016 \pm 0.0001$	$0.3721 \pm 0.0017$	$4.6978 \pm 0.0511$	$18.4056 \pm 0.2089$
16	1	$0.0014 \pm 0.0001$	$0.3856\pm 0.0012$	$3.3656 \pm 0.0348$	$20.2419 \pm 0.1794$

$a_n=1$	$\tilde{a}_n=1$				
$\lambda$	$a_e$	$\tilde{D}_{BC}$	$\tilde{\chi }_{BC}$	$\tilde{\lambda }_{BC}$	$\tilde{\beta }_e$
1.6	0.1	$0.0032\pm 0.0002$	$0.3539 \pm 0.0022$	$1.9091 \pm 0.0474$	$15.0411 \pm 0.2189$
1.6	0.2	$0.0019 \pm 0.0001$	$0.3738 \pm 0.0015$	$1.5556 \pm 0.0365$	$15.4742 \pm 0.1615$
1.6	1	$0.0014 \pm 0.0001$	$0.3819\pm 0.0011$	$1.2633 \pm 0.0324$	$15.8269 \pm 0.1599$
16	0.1	$0.0024\pm 0.0001$	$0.3655 \pm 0.0023$	$2.7258 \pm 0.0537$	$14.0966 \pm 0.2207$
16	0.2	$0.0019 \pm 0.0001$	$0.3844 \pm 0.0016$	$2.0614 \pm 0.0397$	$13.6286 \pm 0.1651$
16	1	$0.0016 \pm 0.0001$	$0.3907\pm 0.0011$	$1.3811 \pm 0.0313$	$14.8300 \pm 0.1516$
